# Supplementary material for: Immune cell dynamics deconvoluted by single-cell RNA sequencing in normothermic machine perfusion of the liver
Source: Nat Commun. 2023 Apr 21;14:2285. doi: 10.1038/s41467-023-37674-8 (PMC10121614; doi:10.1038/s41467-023-37674-8)
Supplement: Supplementary file 1 — Supplementary Information [file 41467_2023_37674_MOESM1_ESM.pdf]

Immune cell dynamics deconvoluted by single-cell RNA sequencing in normothermic machine perfusion of the liver

Hautz T[1]*, Salcher S[2]*, Fodor M[1]*, Sturm G[3], Ebner S[1], Mair A[2], Trebo M[2], Untergasser G[2,4], Sopper S[2], Cardini B[1], Martowicz A[2,4], Hofmann J[1], Daum S[2], Kalb M[2], Resch T[1], Krendl F[1], Weissenbacher A[1], Otarashvili G[1], Obrist P[4], Zelger B[5], Öfner D[1], Trajanoski Z[3], Troppmair J[1], Oberhuber R[1], Pircher A[2], Wolf D[2]**, Schneeberger S[1]**

* These authors contributed equally

** These authors jointly supervised this work.

Affiliations

[1] Department of Visceral, Transplant and Thoracic Surgery, Center of Operative Medicine, organLife Laboratory and D. Swarovski Research Laboratory, Medical University of Innsbruck, Innsbruck, Austria

[2] Department of Internal Medicine V, Hematology and Oncology, Comprehensive Cancer Center Innsbruck (CCCI), Medical University of Innsbruck, Innsbruck, Austria

[3] Institute of Bioinformatics, Biocenter, Medical University of Innsbruck, Innsbruck, Austria

[4] Tyrolpath Obrist Brunhuber GmbH, Zams, Austria

[5] Institute of Pathology, Neuropathology and Molecular Pathology, Medical University of Innsbruck, Austria

Table of contents

Supplementary Figure 1……………………………………………………………………..........4

Supplementary Figure 2………………………………………………………………….......5

Supplementary Figure 3………………………………………………………………….......6

Supplementary Figure 4………………………………………………………………….......7

Supplementary Figure 5………………………………………………………………….......8

Supplementary Figure 6……………………………………………………………………...9

Supplementary Figure 7………………………………………………………………........10

Supplementary Figure 8………………………………………………………………........12

Supplementary Figure 9………………………………………………………………........13

Supplementary Figure 10……………………………………………………………........14

Supplementary Figure 11……………………………………………………………........15

Supplementary Figure 12……………………………………………………………........17

Supplementary Figure 13……………………………………………………………........18

Supplementary Figure 14……………………………………………………………........19

Supplementary Figure 15……………………………………………………………........20

Supplementary Figure 16………………………………………..................................21

Supplementary Figure 17………………………………………..................................22

Supplementary Figure 18………………………………………..................................23

Supplementary Figure 19………………………………………..................................24

Supplementary Figure 20……………………………………..25

Supplementary Table 1………………………...26

Supplementary Table 2……..27

Supplementary Table 3……………..28

Supplementary Table 4…………………..30

Supplementary Table 5…………………..32

Supplementary Table 6…………………..36

Supplementary Table 7…………………..40

Supplementary Table 8…………………..41

Supplementary Table 9…………………..42

Supplementary Figures

Supplementary Figure 1

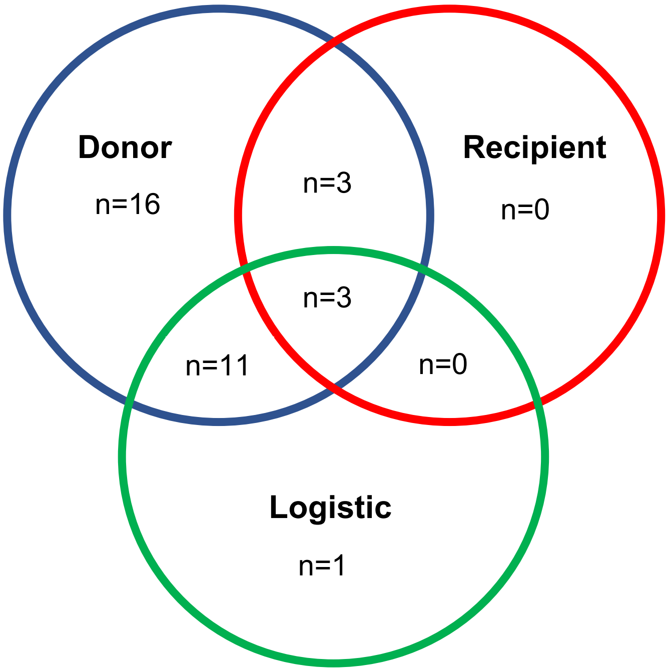

Supplementary Figure 1. Indications for liver normothermic machine perfusion (n=34 donor livers).

Supplementary Figure 2

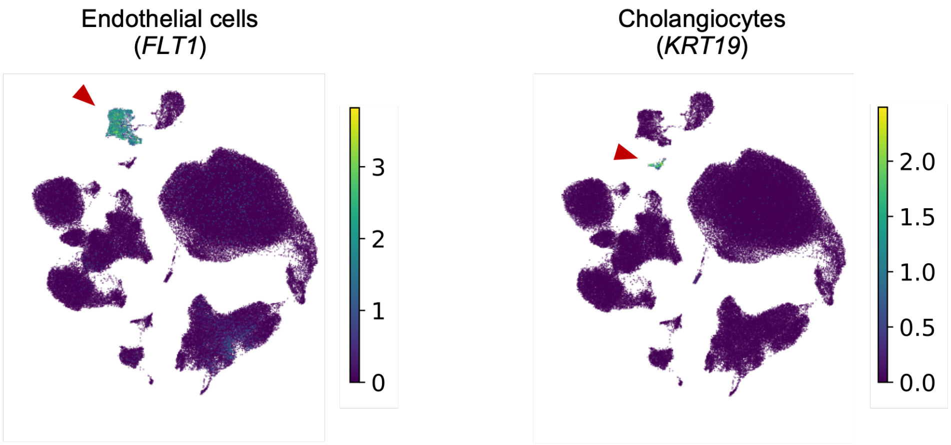

Supplementary Figure 2. Endothelial cells and cholangiocytes in the scRNASeq liver cell atlas. UMAP plot of 118,448 single liver cells color-coded for the expression of marker genes for endothelial cells (*FLT1*) and cholangiocytes (*KRT19*; red arrowheads).

Supplementary Figure 3

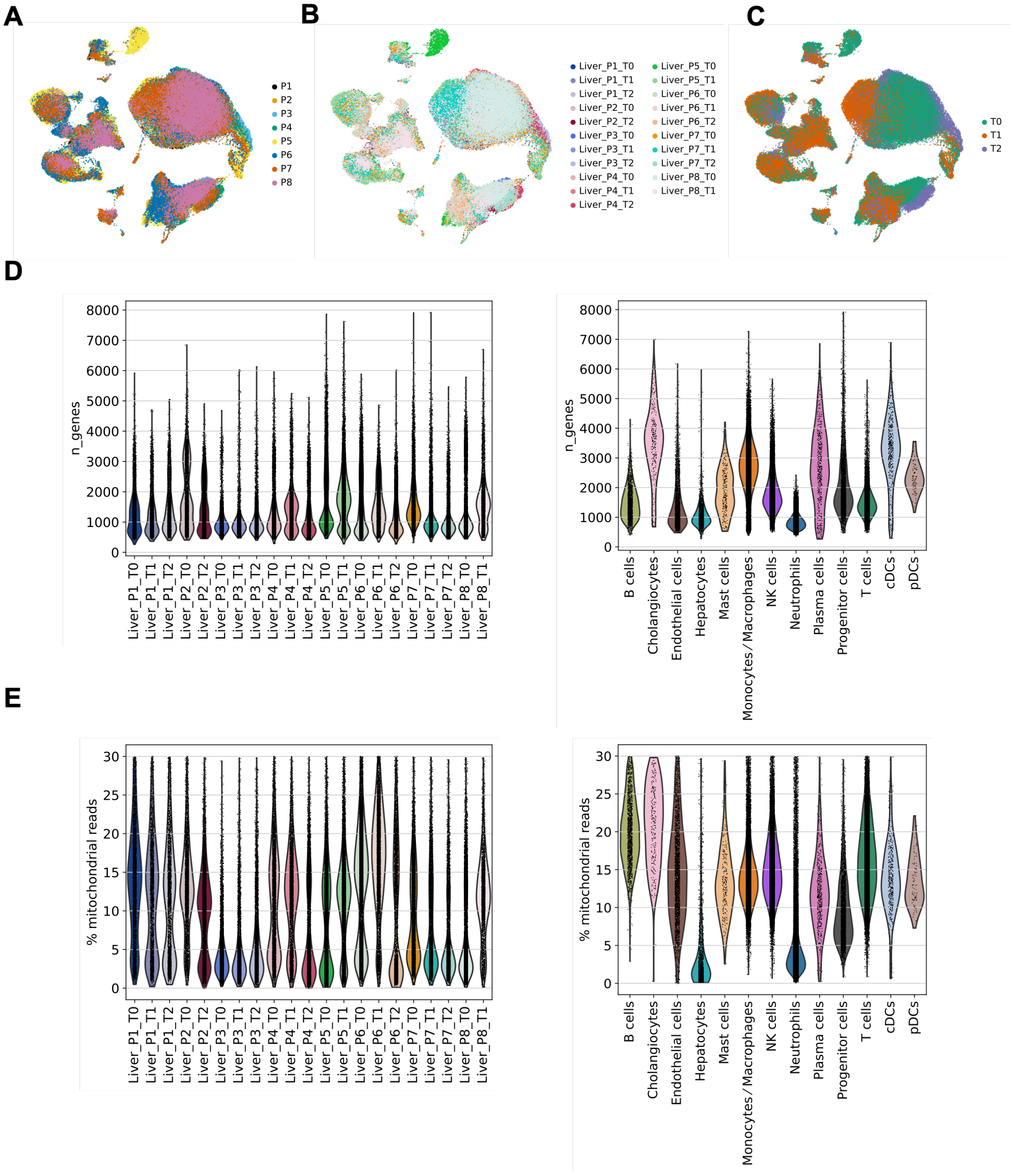

Supplementary Figure 3. scRNASeq dataset composition and QC metrics. (A-C) UMAP plot of 118,448 single cells, color-coded by patient (liver 1-8; Supplementary Figure 2A), by each individual biopsy (liver 1-8; time points T0-T2; Fig. S2B), and by time point [pre NMP (T0), at the end of NMP (T1), after reperfusion (T2); Fig. S2C]. (D) Number of detected genes per cell in each individual biopsy (liver 1-8, time points T0-T2; left figure) and in individual cell types (right figure). (E) Relative abundance of detected mitochondrial transcripts (% mitochondrial UMIs) in each individual biopsy (liver 1-8, time points T0-T2; left figure) and in individual cell types (right figure).

Supplementary Figure 4

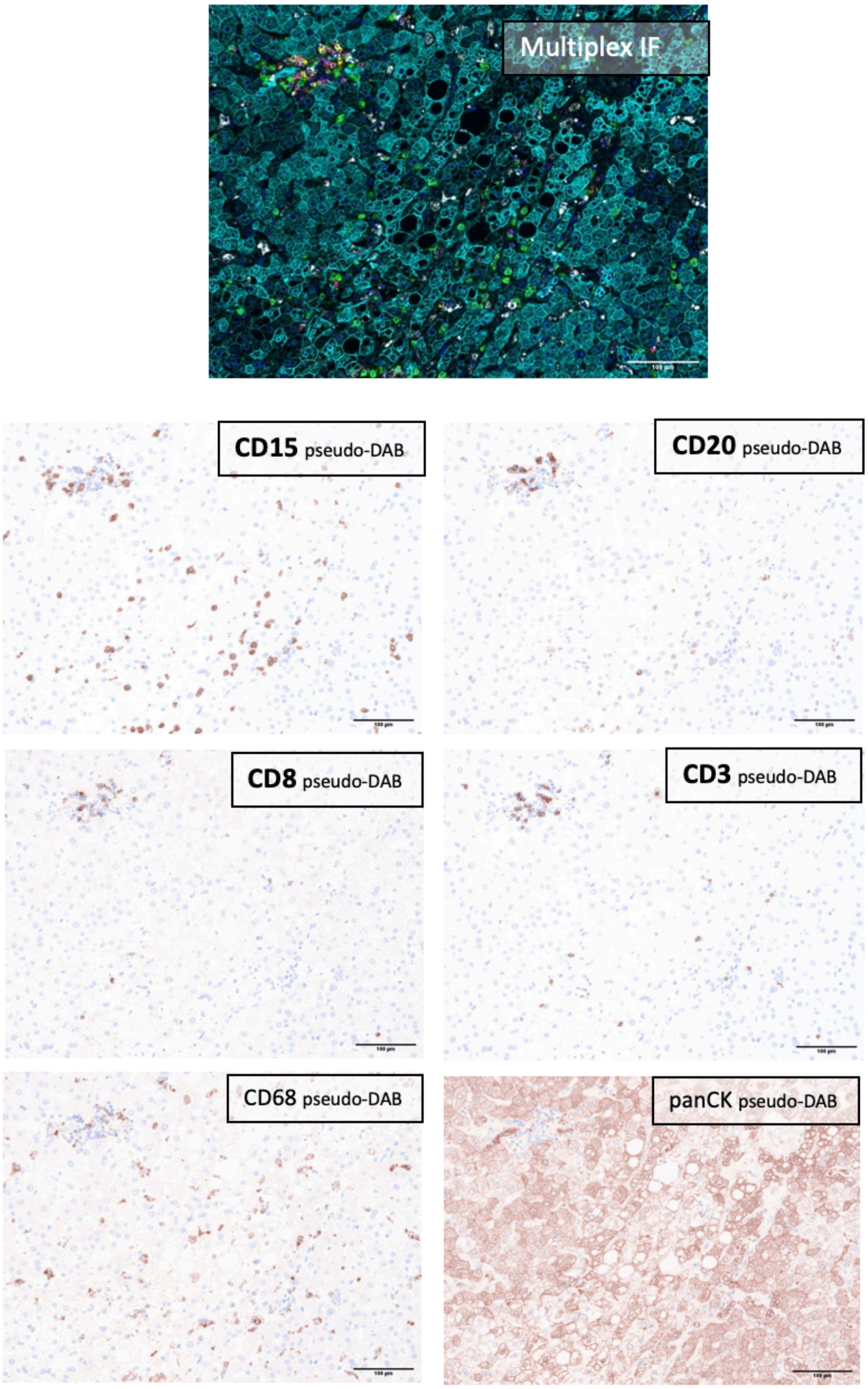

Supplementary Figure 4. Multiplex image viewed in InForm as simulated DAB IHC single stains (single Pathology views, ‘pseudo-DAB‘) of immune-cell characterizing markers in liver biopsies (n=10) pre NMP (T0). Antibodies against CD15 (neutrophils), CD20 (B cells), CD8 (cytotoxic T cells), CD3 (T cells), CD68 (macrophages) and panCK (cytokeratin) were used for characterization of the immune cell repertoire in liver biopsy samples and to establish a panel for multiplex immunofluorescence staining (merge immunofluoresezent image). Images are displayed at 20x magnification (scale bar = 100 µm).

Supplementary Figure 5

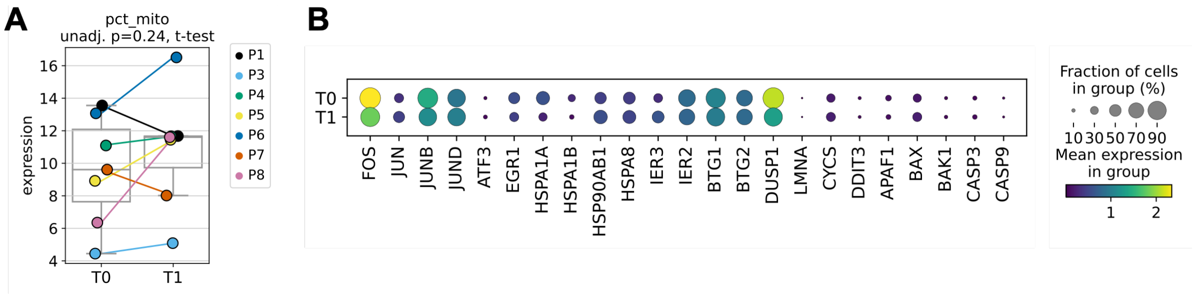

Supplementary Figure 5. Cell stress in liver allografts before and after NMP. (A) Relative abundance of detected mitochondrial transcripts (% mitochondrial reads) in each patient (n=7) pre NMP (T0) and at the end of NMP (T1). The central line denotes the median. Boxes represent the interquartile range (IQR) of the data, whiskers extend to the most extreme data points within 1.5 times the IQR. Two-sided t-test, not adjusted for multiple testing. (B) Expression levels of a set of stress- and apoptosis related genes pre (T0) and at the end of (T1) NMP.

Supplementary Figure 6

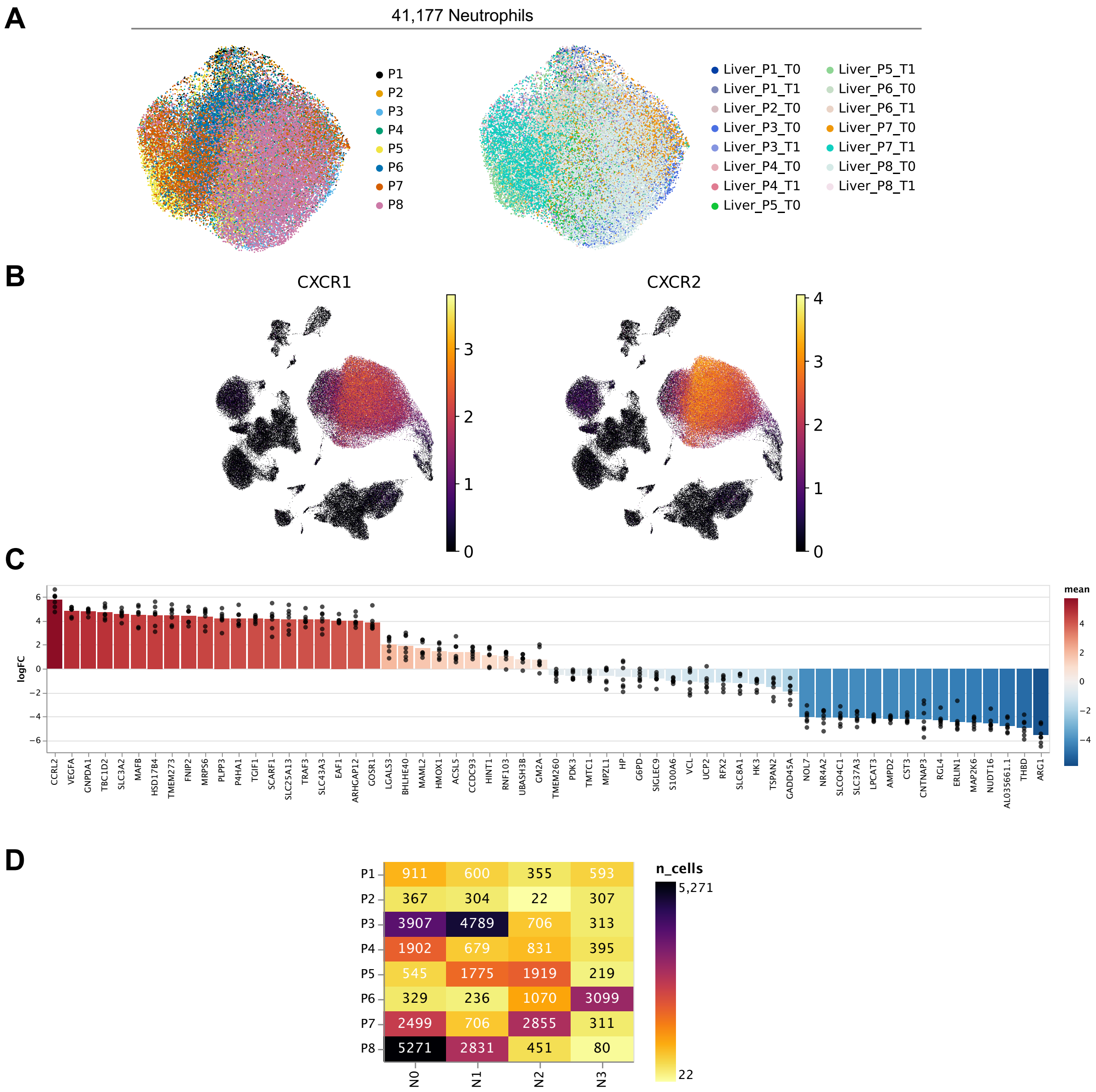

	N0	N1	N2	N3
P1	911	600	355	593
P2	367	304	22	307
P3	3907	4789	706	313
P4	1902	679	831	395
P5	545	1775	1919	219
P6	329	236	1070	3099
P7	2499	706	2855	311
P8	5271	2831	451	80

Supplementary Figure 6. Impact of NMP on neutrophils. (A) UMAP plot of 41,177 neutrophils, color-coded for each individual patient and biopsy (time points T0-T1). (B) UMAP blots depicting *CXCR1* and *CXCR2* gene expression in levels in individual cell clusters. (C) Top differentially expressed genes between pre (T0) and at the end of (T1) NMP. Each dot represents the fold change of an individual patient (n=7). (D) Neutrophil subclusters by individual patient, the heatmap depicts the number of cells per neutrophil cluster.

Supplementary Figure 7

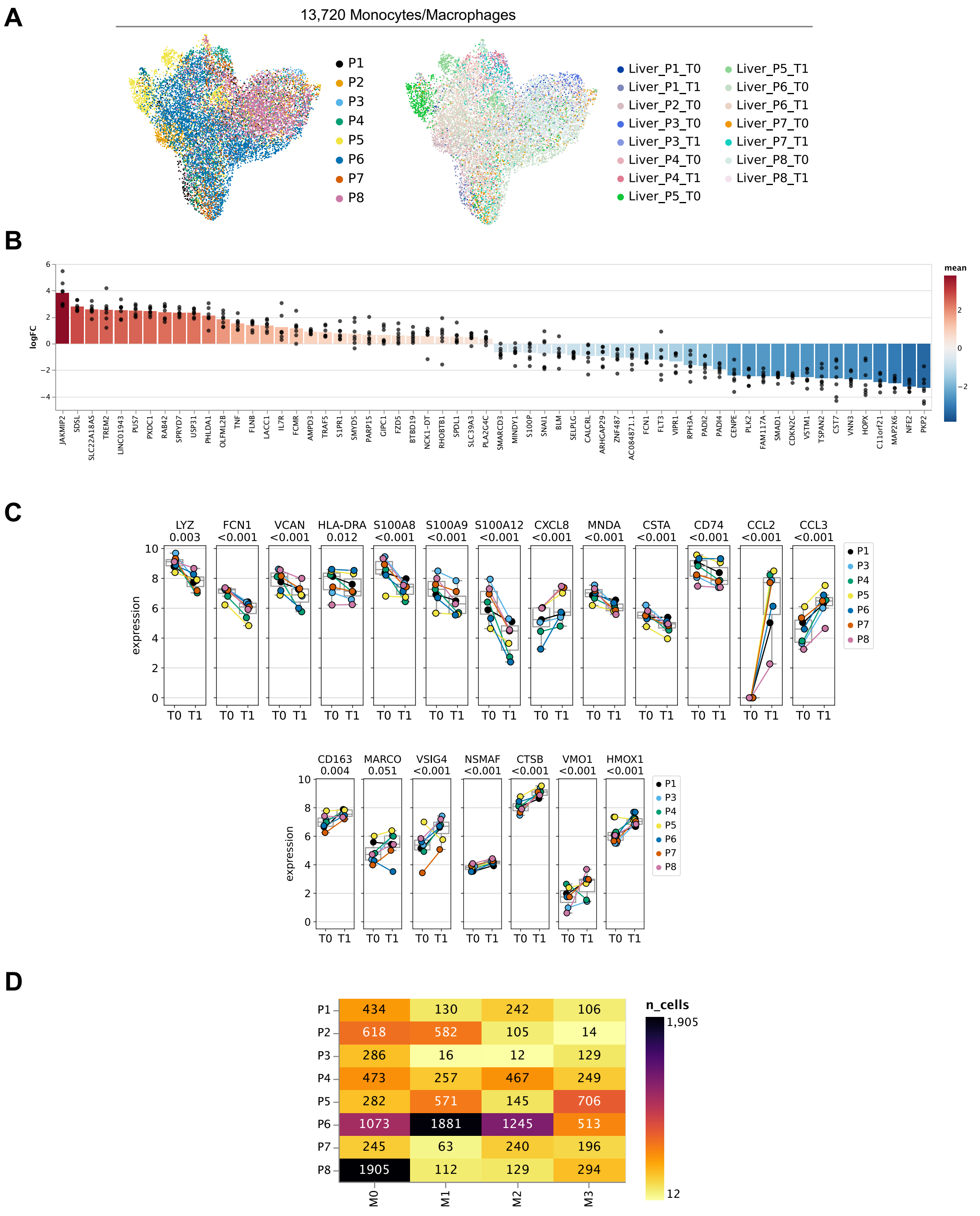

Supplementary Figure 7. Impact of NMP on monocytes/macrophages. (A) UMAP plot of 13,720 monocytes/macrophages, color-coded for each individual patient and biopsy (time points T0-T1). (B) Top differentially expressed genes between pre (T0) and at the end of (T1) NMP. Each dot represents the fold change of an individual patient (n=7). (C) Expression of selected marker genes pre (T0) and at the end of (T1) NMP in individual patients (n=7). Upper panel: inflammatory markers, lower panel: tolerogenic markers. The central line denotes the median. Boxes represent the interquartile range (IQR) of the data, whiskers extend to the most

extreme data points within 1.5 times the IQR. Two-sided DESeq2 Wald-test, p-values are adjusted to false-discovery rate (FDR) using independent hypothesis weighting. (D) Monocytes/macrophages subclusters by individual patient, the heatmap depicts the number of cells per subcluster.

Supplementary Figure 8

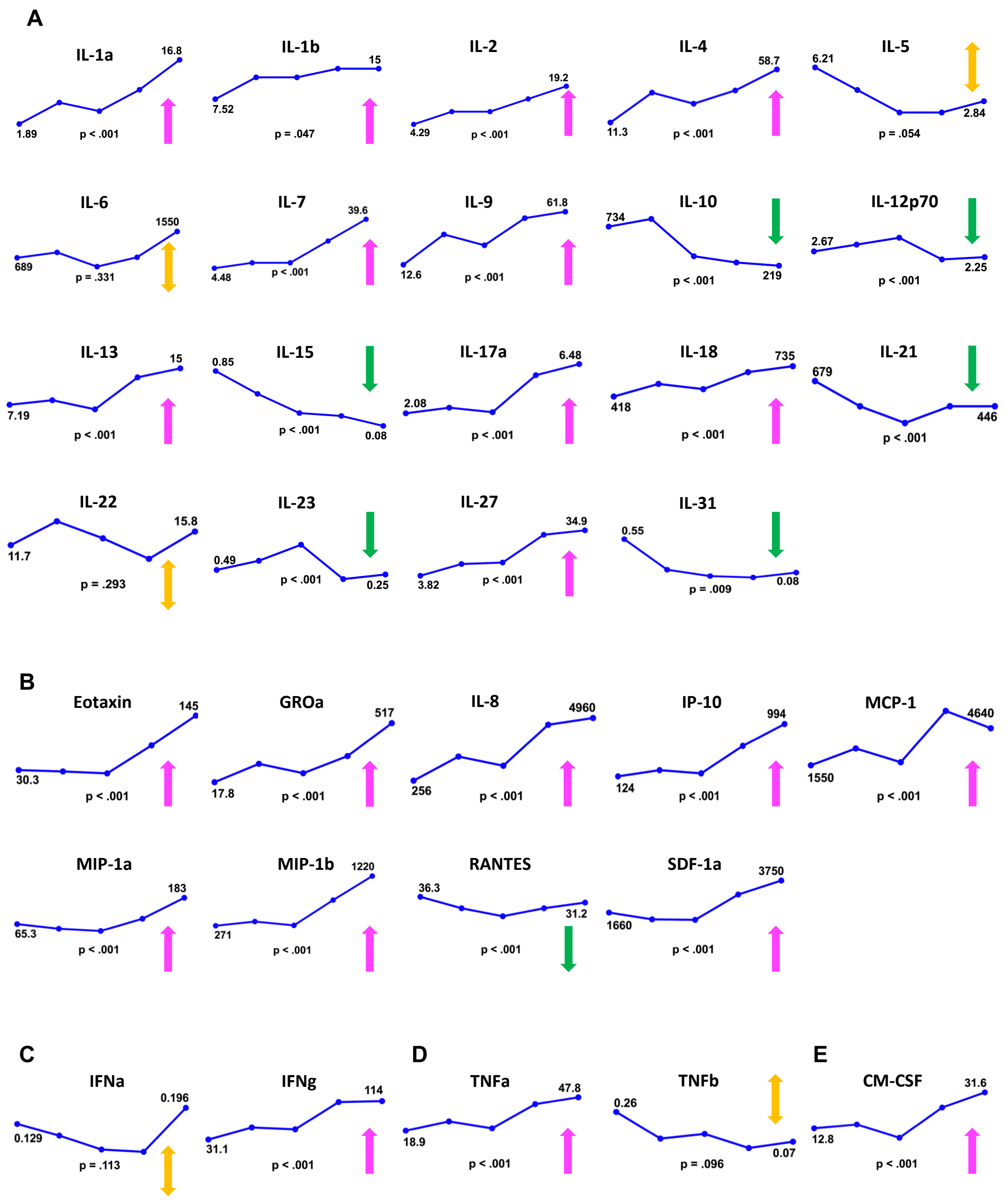

Supplementary Figure 8. Cytokine perfusate levels at 1h, 4h, 6h, 12h and 24h of NMP (n=26 donor livers subjected to NMP). (A) Interleukins (B) Chemokines (C) Interferons (D) Tumour necrosis factors (E) Colony stimulating factors. Magenta arrow: levels significantly increase while NMP, yellow arrow: levels do not alter while NMP, green arrow: levels significantly decrease while NMP. X-axis: time, y-axis: protein levels in pg/ml. The Least Squares Means computed using a linear regression model are shown. The p-values refer to the change over time. Absolute values are shown for 1h and 24h NMP. Samples were measured in duplicate and respective results were averaged. If there was a deviation of more than 20% between the duplicates, the higher value was included in the analysis. Any analyte with a concentration outside the linear range of the Luminex assay was excluded from analysis. N=26 biologically independent samples. Source data are provided as a Source Data file.

Supplementary Figure 9

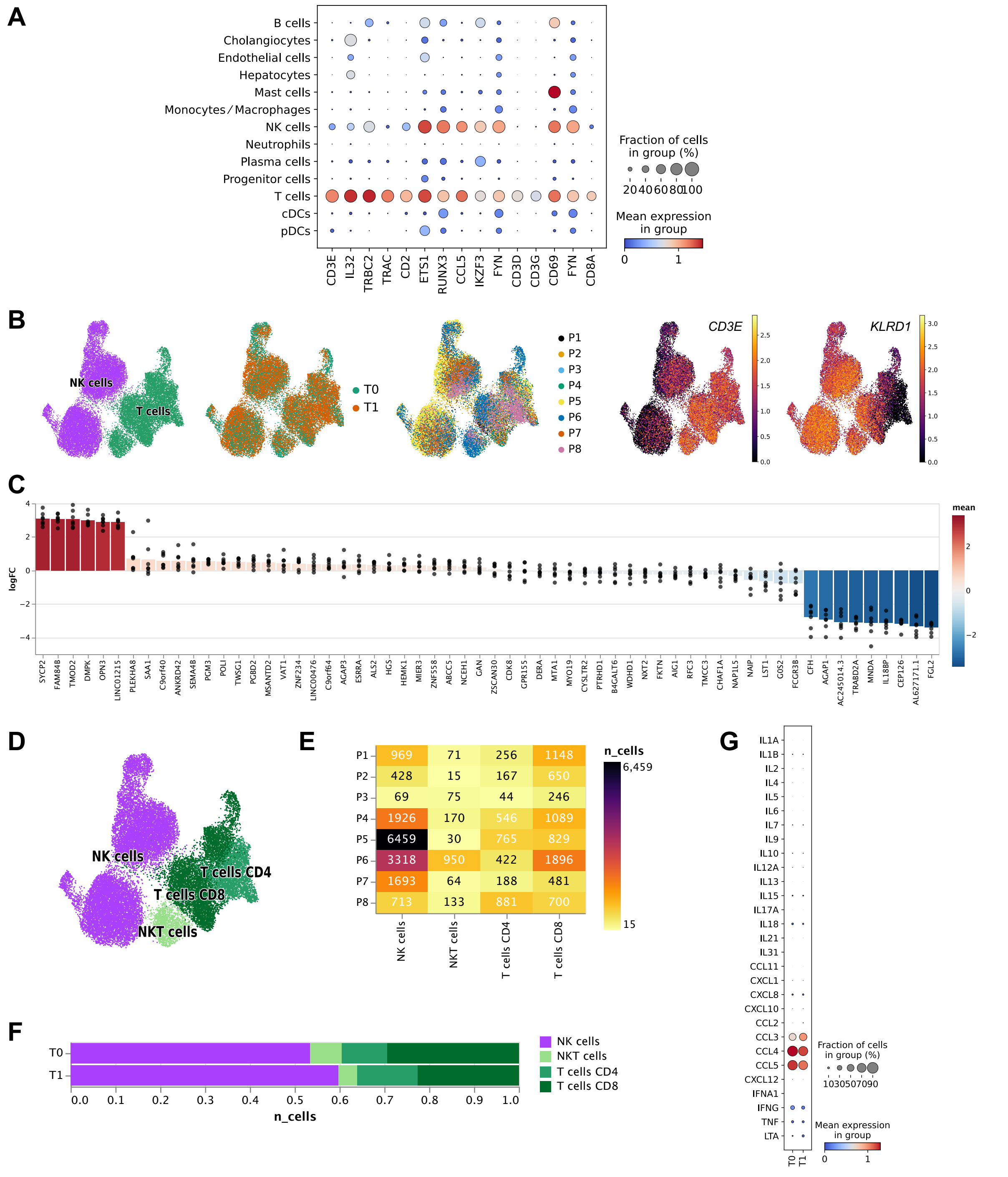

Supplementary Figure 9. Impact of NMP on T cells and NK cells. (A) Gene expression levels of top regulated T/NK cell-specific genes in individual cell types. (B) UMAP plots of 11,816 T cells and 15,575 NK cells, color-coded by time point and by patient, as well as of the *CD3E* and *KLRD1* gene expression. (C) Expression of selected marker genes pre (T0) and at the end of (T1) NMP in individual patients (n=7). (D) UMAP of NK cells, NKT cells, T cells CD8, and T cells CD4. (E) T/NK cell subclusters by individual patient, the heatmap depicts the number of cells per subcluster. (F) Relative proportion of NK cells, NKT cells, T cells CD4 and T cells CD8 pre (T0) and at the end of (T1) NMP. (G) Gene expression levels of indicated interleukins/chemokines in T/NK cells pre (T0) and at the end of NMP (T1) as assessed by scRNASeq in eight donor livers.

Supplementary Figure 10

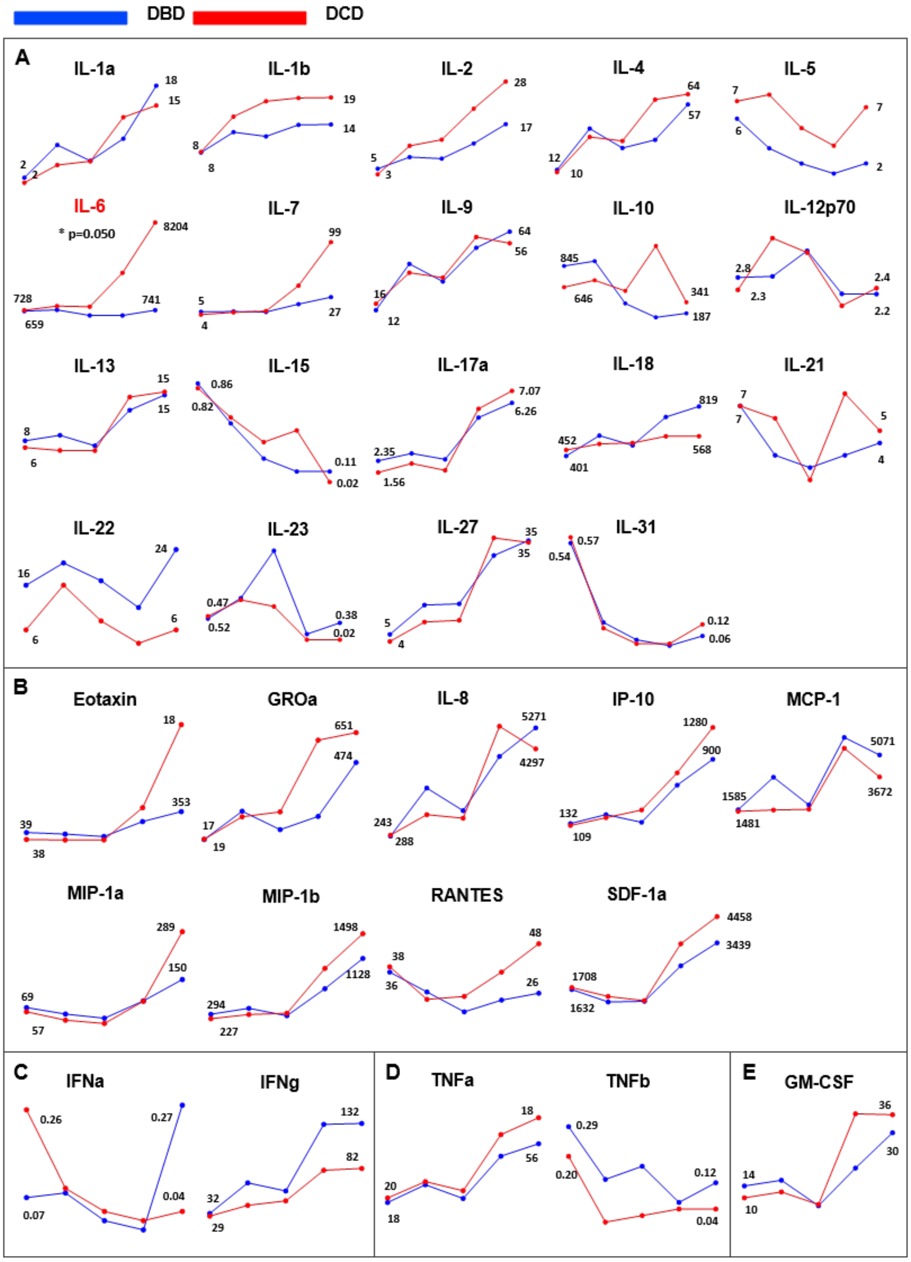

Supplementary Figure 10. Cytokine levels assessed in perfusate samples at various time points during NMP. The perfusate of 26 donor livers subjected to NMP was assessed for its inflammatory profile at 1h, 4h, 6h, 12h and 24h of NMP and differences between DBD (n=18) and DCD (n=8) were calculated. Mediators were grouped for (A) interleukins, (B) chemokines, (C) interferons, (D) tumor necrosis factors and (E) colony stimulating factors. X-axis: time, y-axis: protein levels in pg/ml. The Least Squares Means computed using a linear regression model are shown. The p-values refers to the difference between DBD and DCD. Absolute values are shown for 1h and 24h NMP. P-values are given if a significant change of the analyte was observed over perfusion time, and its name is highlighted in red. Non-significant p-values are presented in Table S 8-9. Samples were measured in duplicates and the respective results were averaged. If there was a deviation of more than 20% between the duplicates, the higher value was included in the analysis. Any analyte with a concentration outside the linear range of the Luminex assay was excluded from analysis. N=26 biologically independent samples. Source data are provided as a Source Data file.

Supplementary Figure 11

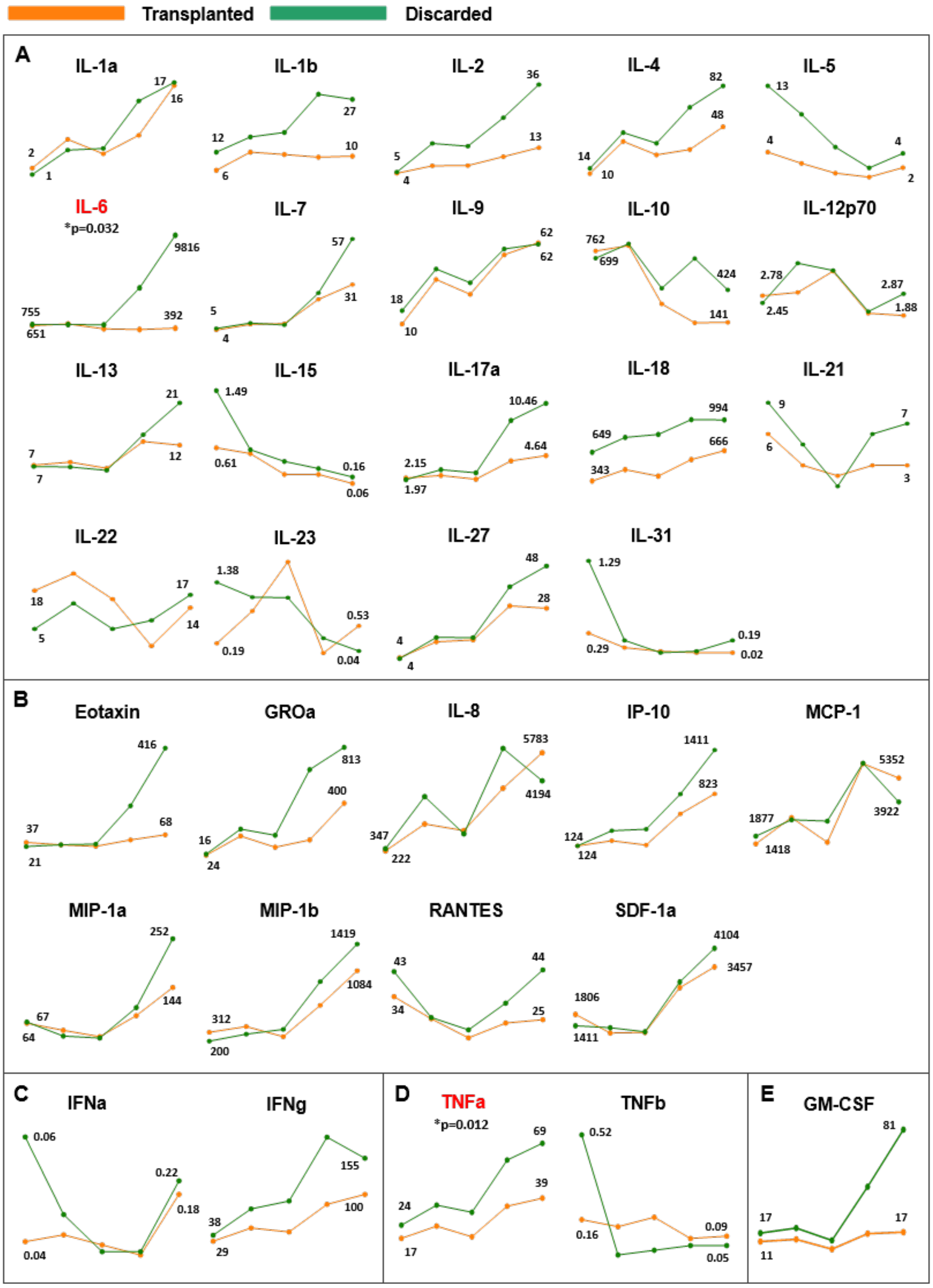

Supplementary Figure 11. Cytokine levels assessed in perfusate samples at various time points during NMP. The perfusate of 26 donor livers subjected to NMP was assessed for its inflammatory profile at 1h, 4h, 6h, 12h and 24h of NMP and differences between transplanted (n=18) and discarded (n=8) livers were calculated. Mediators were grouped for (A) interleukins, (B) chemokines, (C) interferons, (D) tumor necrosis factors and (E) colony stimulating factors. X-axis: time, y-axis: protein levels in pg/ml. The Least Squares Means computed using a linear regression model are shown. The p-values refer to the difference between transplanted and discarded. Absolute values are shown for 1h and 24h NMP. P-values are given if a significant change of the analyte was observed over perfusion time, and its name is highlighted in red. Non-significant p-values are presented in Table S 8-9. Samples were measured in duplicates and the respective results were averaged. If there was a deviation of more than 20% between the duplicates, the higher value was included in the analysis. Any

analyte with a concentration outside the linear range of the Luminex assay was excluded from analysis. N=26 biologically independent samples. Source data are provided as a Source Data file.

Supplementary Figure 12

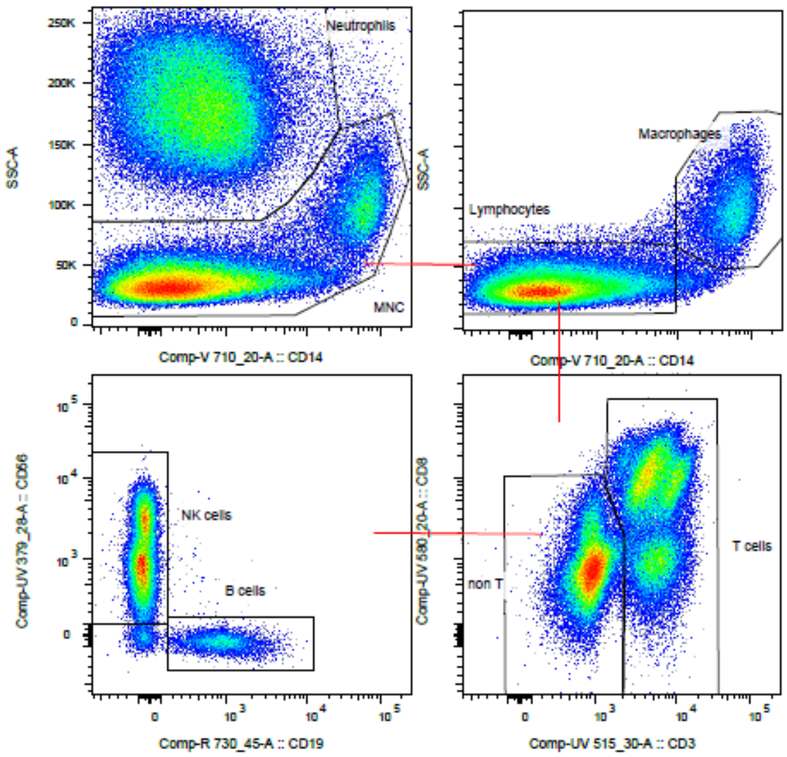

Supplementary Figure 12. Gating strategy for the general immune phenotype panel analysed in liver biopsies [n=7 donor livers at time points pre NMP (T0) and at the end of NMP (T1)]. In initial cleaning steps dead cells, debris and doublets were removed using 7-AAD staining and scatter characteristics. Leukocytes were then defined by CD45 staining end sequentially gated into granulocytes (upper left), macrophages (upper right), T cells (lower right) and B and NK cells (lower left).

Supplementary Figure 13

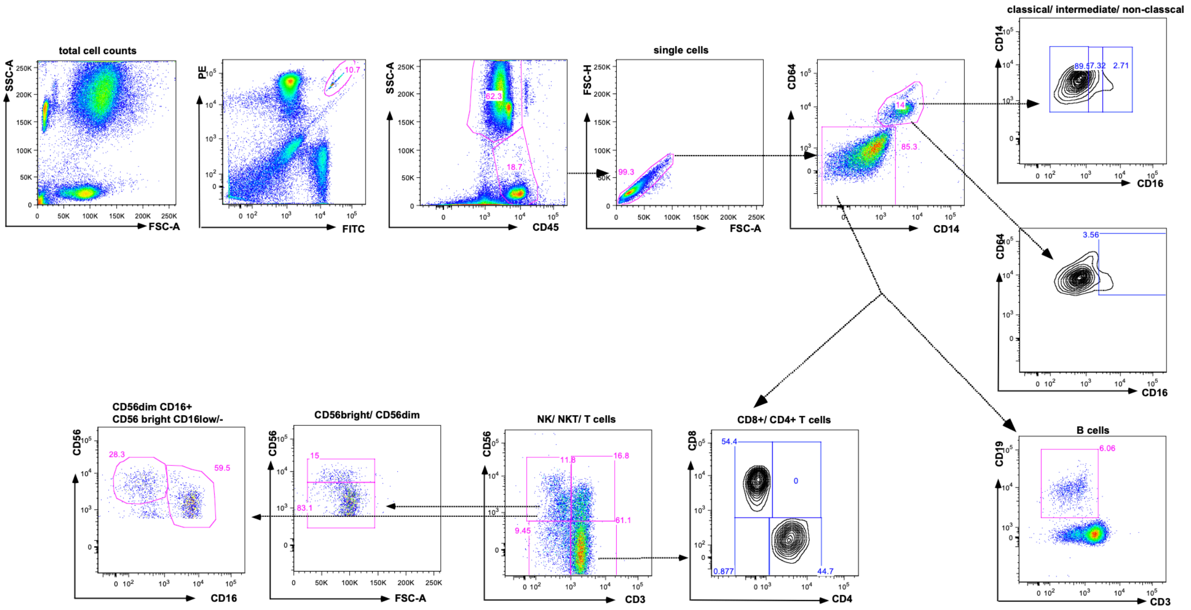

Supplementary Figure 13. Gating strategy for the general immune phenotype panel analyzed in BD TruCount tubes (perfusate of n=26 donor livers subjected to NMP at various time points). Gate for beads were set on an ungated dot plot (FITC vs. PE). Duplets were removed using a FSC-A vs. FSC-H dot pot. Gates for lymphocytes and granulocytes were set on a CD45 vs. SSC-A dot plot. Gating and exclusion of all CD14^{+} monocytes were identified via CD14 versus CD64. The gated CD14^{+} monocytes were used to further discriminate different inflammatory/differentiation stages of monocytes (CD16 versus CD14) resulting in CD14^{++}CD16^{-} classical monocytes, CD14^{++}CD16^{+} and CD14^{+} CD16^{++} monocytes, and CD16 vs CD64 to capture CD16^{+} CD64^{+} monocytes. Lymphocytes (CD14^{-}CD64^{-}) were gated on CD56^{+} NK cells and NKT cells (CD56^{+}CD3^{+}). NK cells were further subdivided into CD56 dim and CD56 high NK cells. Lymphocytes were divided into CD3^{+} T cells (CD56 vs. CD3) – gated T cells were used for identification of CD4^{+} T-cells and CD8^{+} T-cells (CD4 vs. CD8), and the gated lymphocytes were also used for identification of the B cell population (CD19 vs CD3). The total numbers of events for each of these subsets and the number of beads in the TruCount tubes were used to calculate the concentration of the different subsets.

Supplementary Figure 14

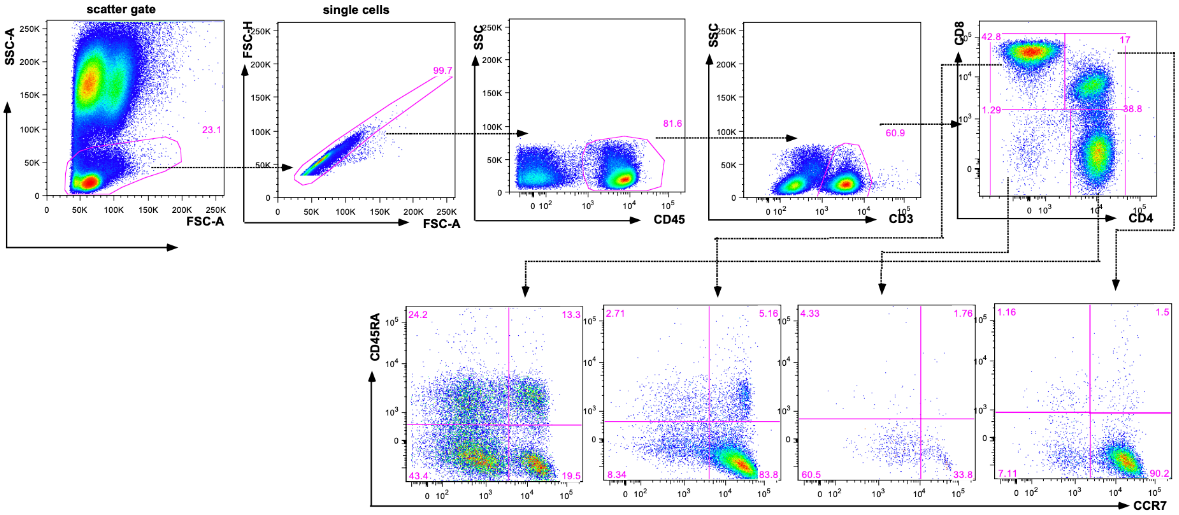

Supplementary Figure 14. Gating strategy for the naive and memory subsets of T cells (perfusate of n=26 donor livers subjected to NMP at various time points). Lymphocytes were gated in a FSC-a vs. SSC-A dot plot and duplets were removed using a FSC-A vs. FSC-H dot pot. $CD45^{+}$ cells and $CD3^{+}$ cells were gated versus SSC-A. $CD3^{+}$ T cells were subdivided into $CD4^{+}$ and $CD8^{+}$ T-cells, double negative and double positive T cells. (CD4 vs. CD8). Naive T cells ($CD45RA^{+}$, $CCR7^{+}$), central memory T cells ($CD45RA^{-}$, $CCR7^{+}$) and effector memory T cells ($CD45RA^{-}$, $CCR7^{-}$) and TEMRA ($CD45RA^{+}$, $CCR7^{-}$) cells were determined.

Supplementary Figure 15

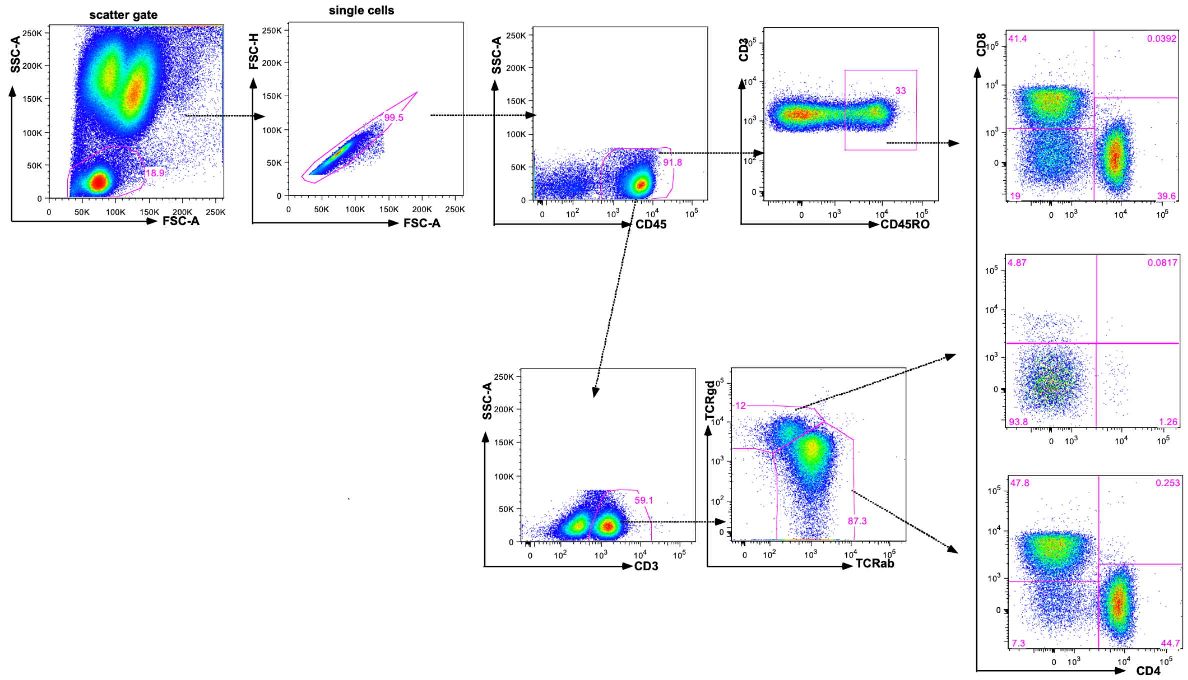

Supplementary Figure 15. Gating strategy for T cells subtypes (perfusate of n=26 donor livers subjected to NMP at various time points). Lymphocytes were gated in a FSC-a vs. SSC-A dot plot and duplets were removed using a FSC-A vs. FSC-H dot pot. CD45$^+$ cells were gated versus SSC-A. CD45$^+$ cells were analyzed for CD3$^+$ CD45RO expression and subdivided into CD4$^+$, CD8$^+$, double negative and double positive CD3$^+$ T cells. Additionally, CD45$^+$ cells were gated in CD3$^+$ T cells vs. SSC-A. CD3$^+$ T cells were subdivided into TCR $\alpha\beta^+$ and TCR $\gamma\delta^+$T cells. These T cells subtypes were further divided into CD4$^+$, CD8$^+$, double negative and double positive T cells.

Supplementary Figure 16

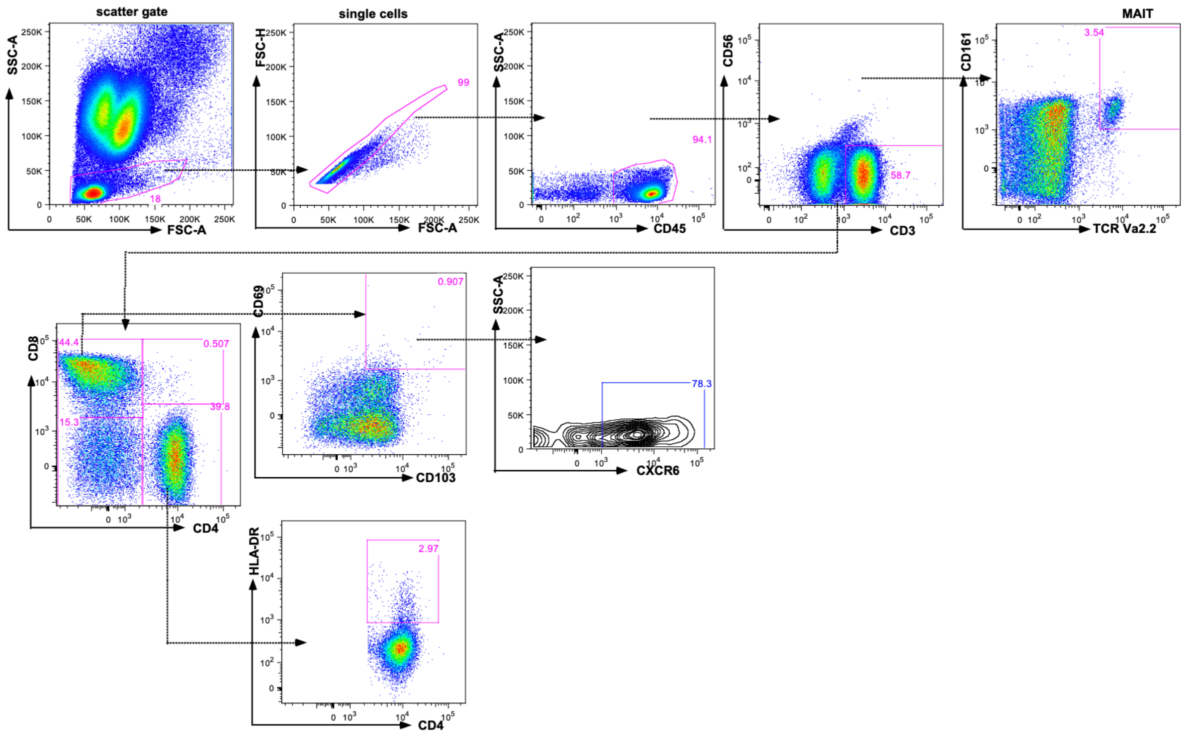

Supplementary Figure 16. Gating strategy for mucosa associated invariant T cells (MAIT) and resident liver T cells (perfusate of n=26 donor livers subjected to NMP at various time points). Lymphocytes were gated in a FSC-a vs. SSC-A dot plot and duplets were removed using a FSC-A vs. FSC-H dot pot. $CD45^+$ cells were gated versus SSC-A. T cells were gated vs CD56. TCR Vα2.2$^+$ $CD161^+$ were defined as MAIT cells. $CD3^+$ T cells were subdivided into $CD4^+$ and $CD8^+$ T cells. Resident $CD8^+$ were defined as $CD103^+$ $CD69^+$ $CXCR6^+$ cells. Gates of CXCR6 were set according to their FMOs. Resident $CD4^+$ T cells were defined as HLA-DR$^+$ cells.

Supplementary Figure 17

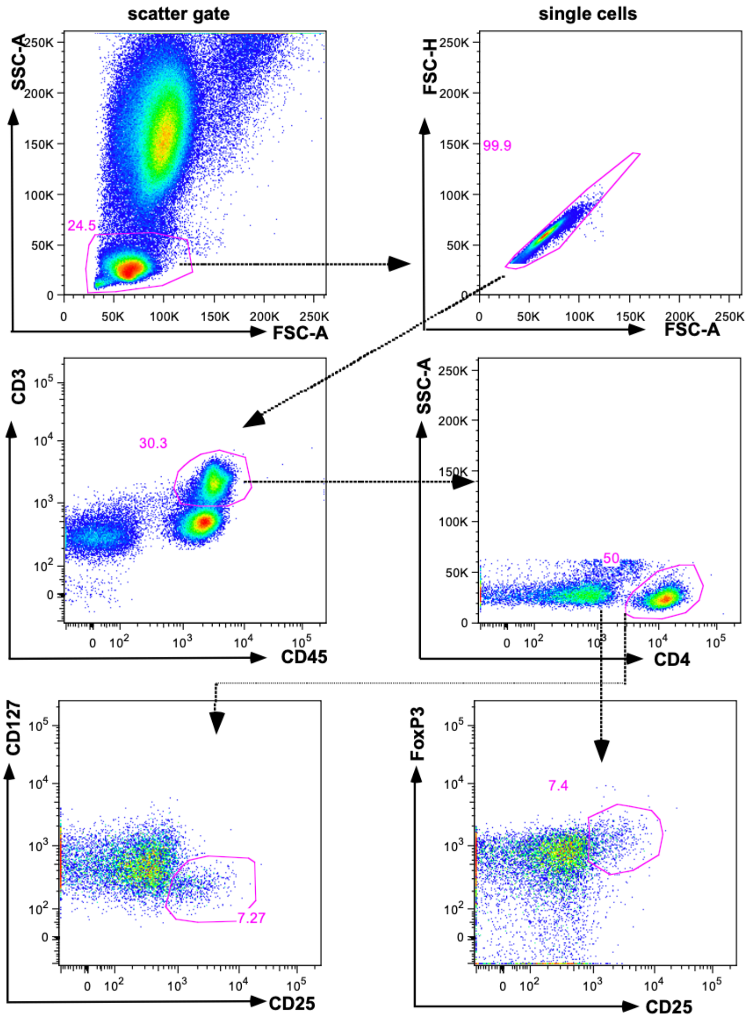

Supplementary Figure 17. Gating strategy for regulatory T cells (perfusate of n=26 donor livers subjected to NMP at various time points). Lymphocytes were gated in a FSC-a vs. SSC-A dot plot and duplets were removed using a FSC-A vs. FSC-H dot pot. $CD3^{+}$ cells were gated versus $CD45^{+}$ cells. $CD3^{+}$ T cells were subdivided into $CD4^{+}$ cells ($CD4^{+}$ vs. SSC-A). Regulatory T cells were determined as $CD25^{+}$ and $FoxP3^{+}$ CD127 dim cells.

Supplementary Figure 18

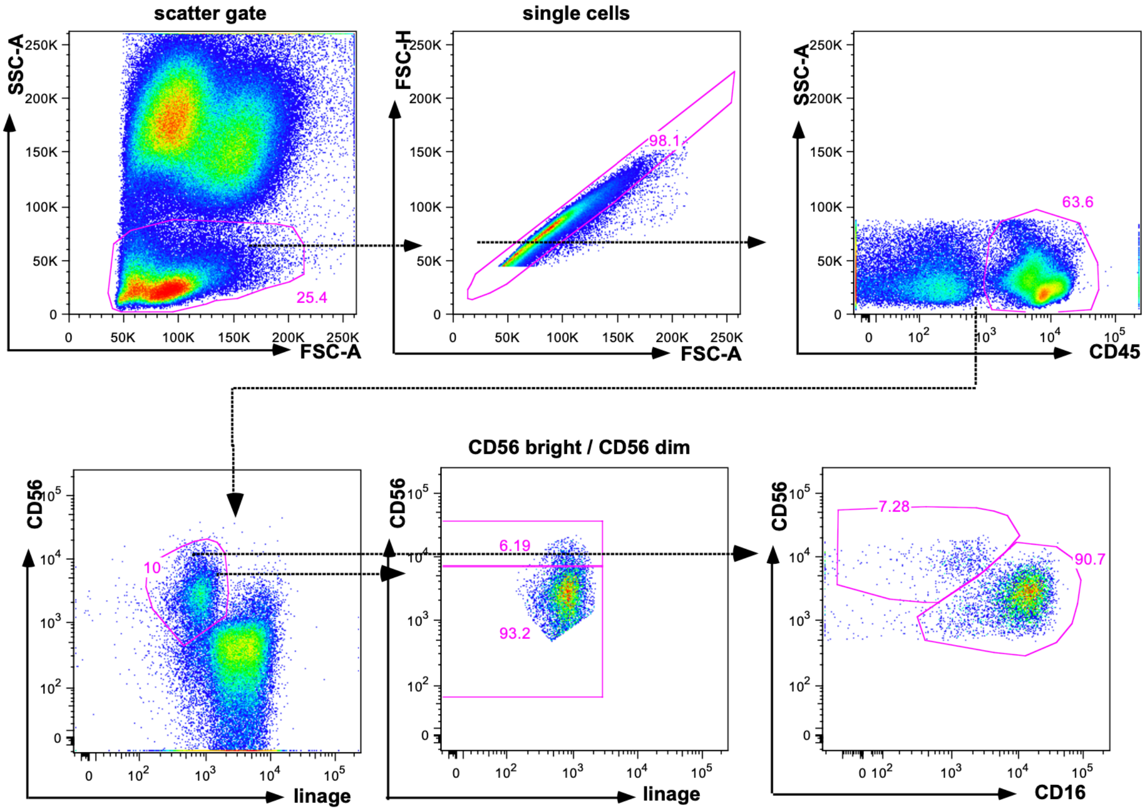

Supplementary Figure 18. Gating strategy for natural killer (NK) cells (perfusate of n=26 donor livers subjected to NMP at various time points). Lymphocytes were gated in a FSC-a vs. SSC-A dot plot and duplets were removed using a FSC-A vs. FSC-H dot pot. CD45^{+} cells were gated versus SSC-A. CD45^{+} cells were further discriminated into CD56^{+} cells and linage - cells (CD3, CD14, CD19). CD56^{+} linage- cells were subdivided into CD56 bright and CD56 dim cells and in CD56^{+} CD16^{-}/dim and CD56^{+} CD16^{+} cells.

Supplementary Figure 19

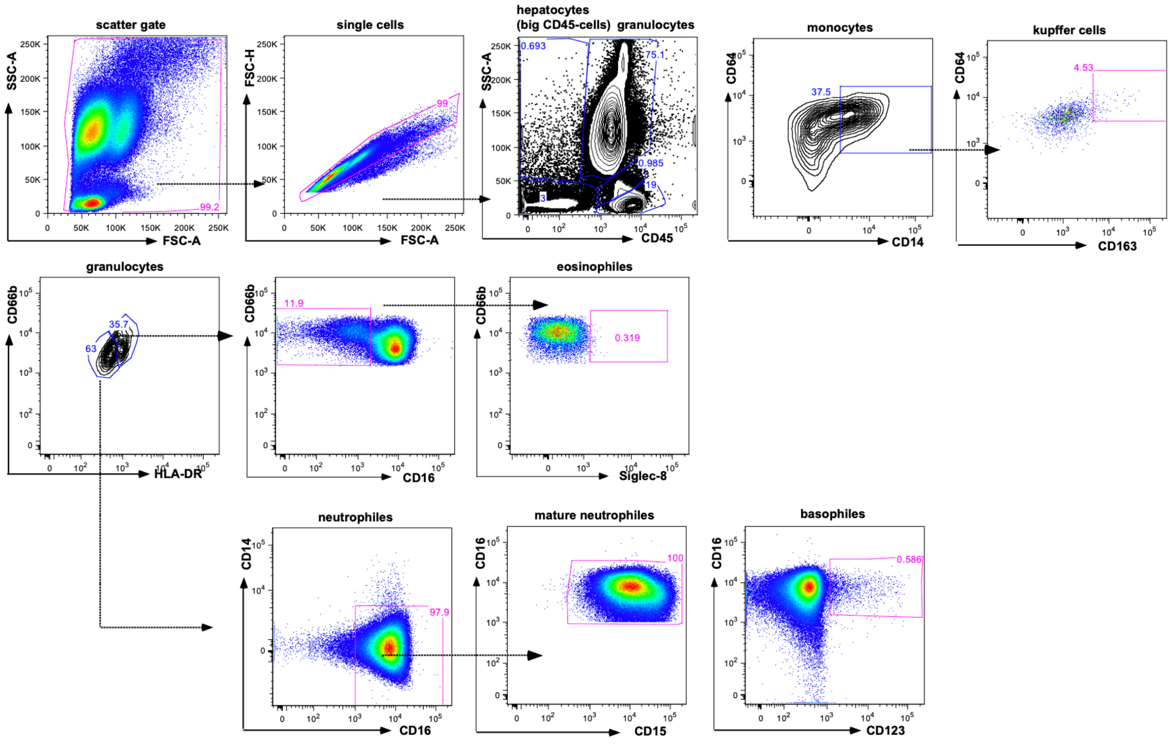

Supplementary Figure 19. Gating strategy for granulocytes, hepatocytes and macrophages/Kupffer cells (perfusate of n=26 donor livers subjected to NMP at various time points). Granulocytes, hepatocytes and macrophages/Kupffer cells were gated in a FSC-A vs. SSC-A dot plot and duplets were removed using a FSC-A vs. FSC-H dot pot. Hepatocytes were defined as big CD45$^-$ cells. Monocytes and macrophages were gated as CD14$^+$ CD64$^+$ cells. CD163$^+$ CD64+ macrophages were subdivided from this population. Granulocytes were divided into HLA-DR low CD66b low and HLA-DR low and CD66b high cells. The latter were defined as eosinophils (Siglec 8$^+$ CD16$^-$). HLA-DR low CD66b low cells were divided into neutrophils (CD16$^+$ CD15$^+$ CD14$^-$) and basophils (CD123$^+$ CD16$^+$).

Supplementary Figure 20

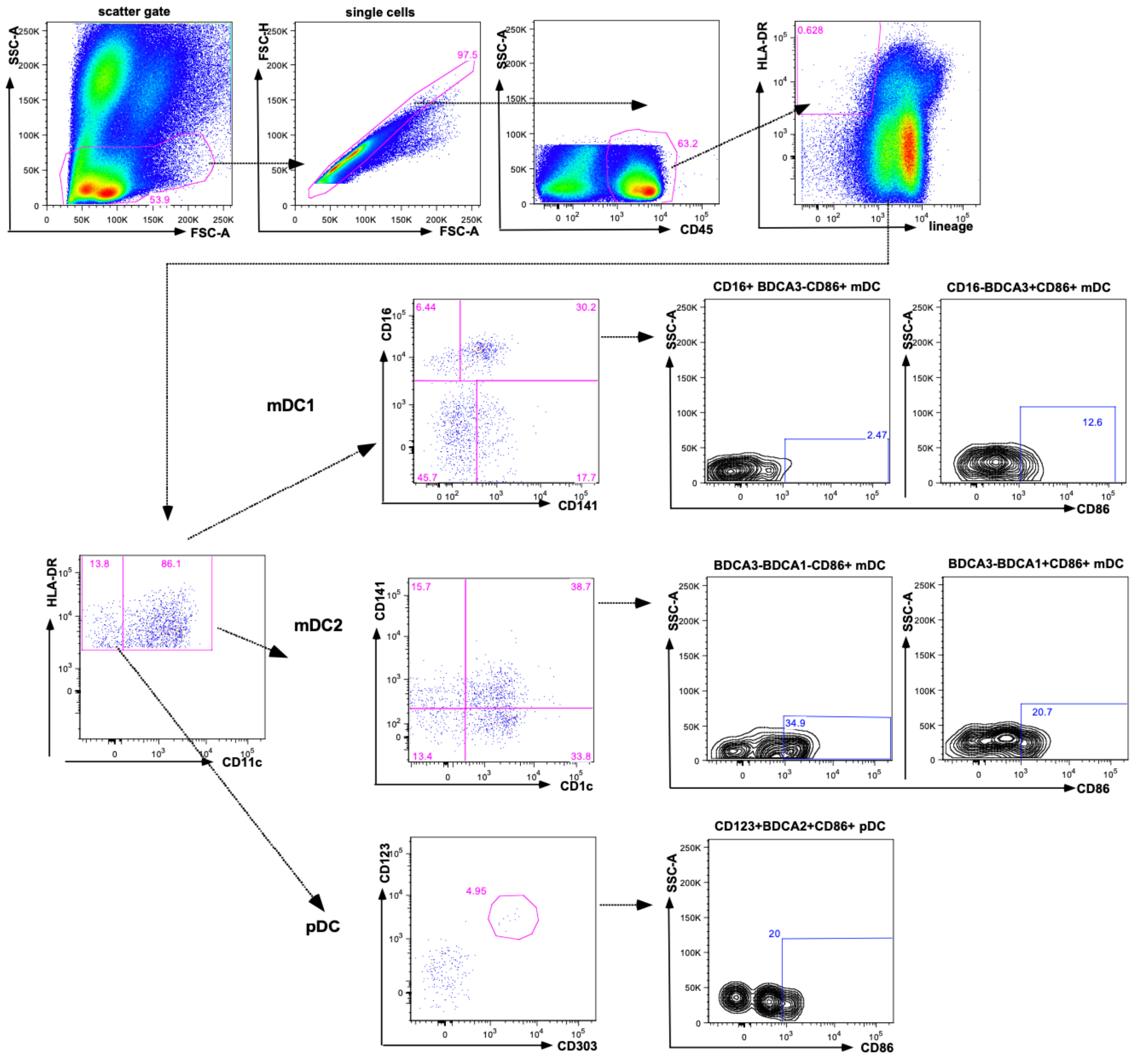

Supplementary Figure 20. Gating strategy for dendritic cells (DCs) (perfusate of n=26 donor livers subjected to NMP at various time points). Lymphocytes were gated in a FSC-a vs. SSC-A dot plot and duplets were removed using a FSC-A vs. FSC-H dot pot. CD45$^+$ cells were gated versus SSC-A. CD45$^+$ cells were further discriminated into HLA-DR$^+$ cells and linage - cells (CD3, CD14, CD19, CD20). These cells were divided into HLA-DR$^+$ CD11c$^-$ cells and HLA-DR$^+$ CD11c$^+$ cells. HLA-DR$^+$ CD11c$^-$ cells were determined as plasmacytoide DCs (pDC) via CD303 and CD123. Activity of pDCs was analyzed with CD86. HLA-DR$^+$ CD11c$^+$ DCs were subdivided into myeloid DC1 (mDC1) via CD141 and CD16 and myeloid DC2 (mDC2) via CD1c and CD141. Activity of subtypes of DCs was determined with CD86. Gates of CD86 were set according to their FMOs.

Supplementary Tables

Supplementary Table 1. Individual donor and preservation data of the study population (n=34)

n	LT	Donor age (y)	Cause of death	Donor type	ECD	Liver DRI	Days ICU donor	Indication for NMP (R/D/L)	CIT > 6h	Total PT (h)
1	Yes	48	CVA	DBD	No	1.82	2	1/1/1	Yes	38.02
2	Yes	56	Trauma	DBD	No	1.31	1	0/1/1	No	14.28
3	No	66	CVA	DBD	Yes	2.25	1	0/1/0	Yes	30.42
4	Yes	74	Circulatory	DBD	Yes	1.90	6	0/1/1	Yes	25.67
5	Yes	53	Circulatory	DBD	Yes	1.88	1	1/1/1	No	10.95
6	Yes	49	CVA	DBD	Yes	1.61	2	0/1/1	Yes	15.63
7	Yes	65	CVA	DBD	Yes	1.77	15	0/1/1	No	17.52
8	Yes	76	CVA	DBD	Yes	2.02	5	0/1/0	Yes	19.22
9	Yes	55	CVA	DBD	No	1.53	3	1/1/0	No	25.98
10	No	31	Circulatory	DCD	Yes	1.77	1	0/1/0	No	26.67
11	Yes	66	Trauma	DBD	Yes	1.28	1	0/0/1	Yes	17.03
12	Yes	73	CVA	DBD	Yes	1.85	1	0/1/1	Yes	27.38
13	No	15	Hypoxia	DBD	Yes	1.20	3	0/1/0	No	23.40
14	Yes	56	CVA	DCD	Yes	3.71	1	0/1/0	Yes	30.65
15	No	53	Trauma	DCD	Yes	2.22	1	0/1/0	Yes	27.65
16	No	59	CVA	DCD	Yes	2.42	8	0/1/0	Yes	27.55
17	Yes	66	CVA	DBD	Yes	1.76	6	0/1/1	No	16.18
18	Yes	28	Trauma	DBD	No	0.90	6	1/1/0	No	26.15
19	No	48	Trauma	DCD	Yes	2.23	4	0/1/0	Yes	29.27
20	Yes	85	CVA	DBD	Yes	1.91	2	0/1/0	No	27.00
21	Yes	68	Trauma	DCD	Yes	1.53	7	1/1/1	No	8.52
22	Yes	62	CVA	DBD	Yes	1.76	2	0/1/0	No	33.57
23	Yes	65	Circulatory	DCD	Yes	2.67	4	0/1/0	Yes	29.83
24	No	79	CVA	DBD	Yes	2.09	5	0/1/0	Yes	115.25
25	No	52	CVA	DCD	Yes	3.66	9	0/1/0	Yes	22.28
26	Yes	75	Trauma	DBD	Yes	1.65	1	1/1/0	Yes	18.03
27	Yes	70	CVA	DBD	Yes	1.86	4	0/1/1	No	16.12
28	Yes	70	Circulatory	DBD	Yes	1.94	4	0/1/1	No	18.22
29	Yes	21	Circulatory	DBD	No	1.59	3	0/1/1	No	25.23
30	Yes	50	CVA	DBD	No	1.38	1	0/1/1	No	26.95
31	No	62	CVA	DBD	Yes	2.06	2	0/1/0	Yes	30.00
32	Yes	72	Trauma	DCD	Yes	1.62	4	0/1/1	No	21.10
33	Yes	84	CVA	DBD	Yes	2.02	2	0/1/0	No	23.83
34	No	54	CVA	DBD	Yes	2.47	2	0/1/0	Yes	20.00

Abbreviations: LT: liver transplantation, NMP: normothermic machine perfusion, y: years, CVA: cerebrovascular accident, ECD: extended criteria donor, DRI: Donor Risk Index, Indication for NMP (R: recipient, D: donor, L: logistic), CIT: cold ischemia time, PT: preservation time, h: hours

Supplementary Table 2. Total cell counts assessed with flow cytometry in perfusate samples at various time points during NMP (n=26 donor livers subjected to NMP).

Cell population	1 h Count/µl average value [95% CI]	4 h Count/µl average value [95% CI]	6 h Count/µl average value [95% CI]	12 h Count/µl average value [95% CI]	24 h Count/µl average value [95% CI]	p-value
**Leucocytes**	2537 [2003, 3215]	2491 [1970, 3149]	2514 [1986, 3181]	2061 [1611, 2635]	1529 [1180, 1982]	<0.001
**Lymphocytes**	450 [346, 585]	385 [297, 498]	402 [309, 521]	324 [246, 426]	234 [174, 313]	<0.001
**T cells**	203 [157, 263]	151 [117, 195]	144 [112, 187]	118 [90, 155]	92 [69, 122]	<0.001
**CD4+ T cells**	93 [73, 120]	79 [61, 101]	77 [60, 99]	63 [48, 82]	44 [33, 59]	<0.001
**CD8+ T cells**	79 [53, 117]	50 [33, 74]	47 [31, 70]	40 [26, 60]	31 [20, 48]	<0.001
**B cells**	52 [38, 71]	52 [38, 71]	60 [44, 82]	53 [38, 73]	35 [24, 49]	0.002
**NK cells**	99 [68, 143]	88 [61, 127]	96 [66, 138]	75 [51, 111]	51 [34, 78]	0.009
**NKT cells**	26 [17, 38]	19 [13, 29]	19 [13, 28]	16 [10, 24]	12 [7, 18]	0.003
**Monocytes**	37 [26, 54]	52 [36, 76]	64 [44, 93]	39 [27, 58]	30 [20, 46]	<0.001
**Granulocytes**	1947 [1510, 2511]	1988 [1544, 2559]	1972 [1530, 2540]	1643 [1262, 2139]	1219 [924, 1607]	<0.001

Data are shown as average values and 95% confidence intervals (in brackets), estimated by linear mixed effect model for repeated measures. The p-values refer to the change over time and were assessed using ANOVA.

Supplementary Table 3. Cell frequencies assessed with flow cytometry in perfusate samples at various time points during NMP (n=26 donor livers subjected to NMP).

Cell population	1 h % (average value [95% CI])	4 h % (average value [95% CI])	6 h % (average value [95% CI])	12 h % (average value [95% CI])	24 h % (average value [95% CI])	p-value
**T cells**	47 [41, 52]	42 [37, 48]	39 [33, 44]	39 [33, 45]	42 [36, 48]	0.007
**CD4+ T cells**	48 [42, 54]	55 [49, 61]	55 [49, 61]	55 [49, 62]	52 [45, 58]	<0.001
**CD8+ T cells**	44 [37, 50]	38 [31, 44]	37 [31, 44]	38 [31, 45]	41 [33, 48]	<0.001
**Foxp3+ T cells**	2.3 [1.6, 3.3]	2.5 [1.7, 3.5]	2.8 [1.9, 3.9]	3.7 [2.6, 5.2]	4.7 [3.3, 6.6]	<0.001
**γδ T cells**	2.7 [1.4, 4.7]	2.3 [1.1, 4.0]	2.2 [1.1, 3.9]	2.5 [1.3, 4.3]	2.4 [1.2, 4.3]	0.749
**DN T cells**	6.6 [4.7, 9.2]	6.3 [4.5, 8.8]	6.3 [4.5, 8.8]	6.8 [4.8, 9.5]	7.9 [5.5, 11.3]	0.518
**MAIT cells**	1.4 [1.1, 1.7]	1.4 [1.1, 1.7]	1.4 [1.1, 1.7]	1.5 [1.1, 1.8]	1.4 [1.1, 1.8]	0.997
**Dendritic cells**	0.86 [0.53, 1.27]	0.67 [0.36, 1.04]	0.62 [0.33, 0.98]	0.83 [0.48, 1.27]	0.83 [0.43, 1.35]	0.530
**B cells**	13 [10, 16]	15 [12, 18]	17 [14, 20]	19 [16, 22]	16 [13, 20]	0.001
**NK cells**	22 [18, 27]	23 [19, 28]	24 [19, 29]	23 [19, 29]	22 [17, 28]	0.887
**NKT cells**	7.5 [5.8, 9.2]	6.4 [4.7, 8.1]	5.9 [4.2, 7.7]	6.3 [4.5, 8.1]	6.0 [4.2, 7.9]	0.103
**Monocytes**	7 [5, 10]	11 [8, 15]	13 [9, 17]	10 [7, 14]	10 [7, 15]	0.001
**Granulocytes**	60 [54, 65]	62 [57, 68]	62 [57, 68]	61 [56, 67]	58 [52, 63]	0.122
**Kupffer cells**	5.6 [3.4, 8.8]	4.0 [2.4, 6.4]	4.0 [2.4, 6.5]	6.7 [4.1, 10.7]	11.9 [7.1, 19.5]	0.099
**Hepatocytes**	0.86 [0.64, 1.10]	0.80 [0.60, 1.03]	0.95 [0.72, 1.20]	0.67 [0.46, 0.90]	1.10 [0.80, 1.45]	0.001
**CD4+ cmT cells**	15.98 [11.25, 20.72]	18.88 [14.15, 23.60]	20.17 [15.44, 24.90]	19.36 [14.59, 24.13]	15.60 [10.70, 20.50]	<0.001
**CD4+ emT cells**	38.90 [32.73, 46.21]	31.57 [26.57, 37.47]	31.12 [26.17, 36.96]	27.49 [22.94, 32.89]	28.22 [23.11, 34.40]	<0.001
**CD4+ TEMRA T cells**	18.52 [12.07, 28.17]	12.77 [8.23, 19.53]	10.61 [6.78, 16.33]	10.50 [6.65, 16.30]	12.12 [7.53, 19.20]	<0.001
**CD8+ em T cells**	4.12 [2.60, 6.27]	5.26 [3.42, 7.86]	5.87 [3.84, 8.75]	5.30 [3.39, 8.04]	4.31 [2.58, 6.87]	0.141
**CD8+ em T cells**	49.81 [40.01, 59.61]	48.18 [38.42, 57.94]	47.75 [37.97, 57.53]	45.88 [35.91, 55.85]	48.43 [37.90, 58.96]	0.746
**CD8+ TEMRA T cells**	31.34 [23.62, 41.47]	24.74 [18.64, 32.74]	25.70 [19.36, 34.03]	23.95 [17.87, 32.01]	22.35 [16.24, 30.63]	0.024

**CD4+CD28- T cells**	47 [34, 60]	51 [38, 64]	51 [38, 64]	53 [40, 66]	54 [41, 67]	0.096
**CD8+CD28- T cells**	72 [65, 80]	74 [66, 81]	74 [66, 81]	74 [66, 81]	74 [66, 82]	0.946
**Myeloid DC 1**	55.01 [44.79, 65.23]	58.25 [48.12, 68.38]	57.85 [47.47, 68.22]	56.58 [45.88, 67.28]	48.88 [37.08, 60.69]	0.461
**Activated mDC1**	18.15 [10.36, 31.28]	10.66 [5.91, 18.65]	9.64 [5.29, 16.99]	7.51 [3.99, 13.51]	5.24 [2.57, 9.89]	<0.001
**Myeloid DC 2**	8.80 [5.31, 14.22]	16.18 [10.11, 25.57]	14.01 [8.69, 22.25]	17.96 [10.93, 29.15]	18.62 [10.83, 31.56]	0.011
**Activated mDC2**	39.27 [26.82, 51.73]	46.05 [33.62, 58.48]	44.77 [32.33, 57.21]	51.80 [39.19, 64.41]	50.13 [37.22, 63.04]	<0.001
**Plasmacytoid DC**	28.45 [16.49, 40.40]	30.39 [18.44, 42.34]	26.58 [13.78, 39.37]	28.83 [14.03, 43.62]	12.62 [4.53, 29.78]	0.367
**Activated pDC**	1.85 [0.58, 4.12]	1.03 [0.13, 2.65]	0.58 [0.15, 1.94]	0.72 [0.14, 2.43]	0.94 [0.11, 3.23]	0.283
**CD56+ dim CD16+ NK cells**	94.21 [96.11, 91.58]	94.46 [96.28, 91.93]	95.16 [96.79, 92.90]	94.33 [96.25, 91.65]	93.51 [95.74, 90.34]	0.466
**CD56+ bright CD16- NK cells**	2.46 [1.66, 3.50]	2.30 [1.55, 3.29]	1.96 [1.28, 2.84]	1.85 [1.17, 2.75]	2.21 [1.41, 3.28]	0.396
**CD56+ bright CD16+ dim NK cells**	3.43 [2.37, 4.82]	3.13 [2.15, 4.42]	2.49 [1.66, 3.58]	2.60 [1.72, 3.76]	2.85 [1.88, 4.15]	0.067
**Classical monocytes**	64.29 [56.56, 72.01]	59.67 [52.03, 67.32]	55.11 [47.43, 62.78]	55.78 [47.68, 63.88]	46.97 [38.36, 55.57]	<0.001
**Intermediate monocytes**	27.67 [20.04, 35.30]	31.33 [23.76, 38.89]	33.62 [26.03, 41.21]	33.93 [25.97, 41.88]	40.00 [31.60, 48.40]	0.021
**Non classical monocytes**	5.57 [3.66, 8.26]	5.72 [3.78, 8.44]	7.39 [4.97, 10.80]	6.21 [4.04, 9.32]	7.85 [5.05, 11.95]	0.276
**Neutrophils**	93.46 [96.30, 88.90]	94.08 [96.68, 89.92]	94.39 [96.88, 90.41]	93.43 [96.31, 88.78]	92.59 [95.87, 87.22]	0.379
**Basophils**	0.23 [0.13, 0.34]	0.25 [0.15, 0.37]	0.28 [0.17, 0.39]	0.26 [0.15, 0.38]	0.28 [0.17, 0.40]	0.730
**Eosinophils**	0.38 [0.20, 0.59]	0.32 [0.15, 0.51]	0.32 [0.15, 0.52]	0.29 [0.11, 0.49]	0.29 [0.10, 0.51]	0.756

Data are shown as average values and 95% confidence intervals (in brackets), estimated by linear mixed effect model for repeated measures. The p-values refer to the change over time and were assessed using ANOVA.

Supplementary Table 4. Cytokine levels assessed in perfusate samples at various time points during NMP (n=26 donor livers subjected to NMP).

Cytokine	1 h (pg/ml) average value [95% CI]	4 h (pg/ml) average value [95% CI]	6 h (pg/ml) average value [95% CI]	12 h (pg/ml) average value [95% CI]	24 h (pg/ml) average value [95% CI]	p-value
**Interleukins**						
**IL-1a**	2 [1, 4]	7 [4, 11]	5 [3, 9]	10 [6, 18]	17 [10, 29]	<0.001
**IL-1b**	8 [5, 12]	13 [8, 20]	13 [8, 20]	15 [9, 26]	15 [9, 25]	0.047
**IL-2**	4 [2, 7]	9 [5, 14]	9 [5, 14]	14 [8, 24]	19 [11, 33]	<0.001
**IL-4**	11 [7, 17]	38 [26, 56]	28 [19, 41]	40 [25, 64]	59 [38, 91]	<0.001
**IL-5**	6 [3, 12]	4 [2, 9]	2 [1, 5]	2 [0, 4]	3 [1, 7]	0.054
**IL-6**	689 [286, 1658]	866 [346, 2162]	400 [159, 1004]	706 [228, 2181]	1551 [537, 4473]	0.331
**IL-7**	4 [3, 6]	8 [6, 10]	8 [6, 10]	24 [18, 31]	40 [31, 51]	<0.001
**IL-9**	13 [6, 19]	41 [34, 47]	31 [25, 38]	56 [47, 64]	62 [54, 70]	<0.001
**IL-10**	734 [407, 1322]	835 [457, 1528]	344 [179, 660]	261 [131, 516]	219 [113, 423]	<0.001
**IL12p70**	2.7 [2.2, 3.2]	3.3 [2.8, 3.9]	3.9 [3.3, 4.6]	2.0 [1.5, 2.6]	2.2 [1.8, 2.8]	<0.001
**IL-13**	7 [6, 9]	8 [6, 10]	6 [5, 8]	13 [10, 18]	15 [11, 20]	<0.001
**IL-15**	0.85 [0.45, 1.35]	0.53 [0.21, 0.95]	0.26 [0.02, 0.60]	0.22 [0.09, 0.63]	0.08 [0.18, 0.43]	<0.001
**IL-17a**	2.1 [1.2, 3.4]	2.6 [1.6, 4.2]	2.2 [1.3, 3.6]	5.5 [3.3, 8.7]	6.5 [4.1, 10.1]	<0.001
**IL-18**	418 [298, 585]	550 [394, 769]	495 [353, 694]	673 [468, 967]	735 [515, 1050]	<0.001
**IL-21**	7 [2, 17]	4 [1, 10]	2 [0, 5]	4 [1, 11]	4 [1, 12]	<0.001
**IL-22**	12 [5, 28]	19 [8, 45]	14 [5, 32]	8 [2, 21]	16 [6, 40]	0.293
**IL-23**	0.5 [0.1, 1.0]	0.9 [0.4, 1.6]	1.6 [0.9, 2.5]	0.1 [0.3, 0.6]	0.3 [0.1, 0.8]	<.001
**IL-27**	4 [2, 7]	12 [7, 21]	13 [7, 22]	32 [17, 61]	35 [19, 63]	<0.001
**IL-31**	0.55 [0.27, 0.90]	0.12 [0.08, 0.36]	0.03 [0.16, 0.26]	0.01 [0.24, 0.27]	0.08 [0.15, 0.37]	0.009
**Chemokines**						
**Eotaxin**	30 [20, 46]	27 [18, 41]	23 [15, 35]	82 [49, 136]	145 [89, 234]	<0.001

**GROa**	18<br>[10, 30]	173<br>[105, 285]	94<br>[55, 159]	239<br>[130, 440]	517<br>[290, 921]	<0.001
**IL-8**	256<br>[167, 392]	2065<br>[1270, 3358]	1372<br>[675, 2785]	4453<br>[2478, 7999]	4958<br>[2921, 8414]	<0.001
**IP-10**	124<br>[82, 187]	227<br>[151, 340]	174<br>[114, 265]	628<br>[379, 1040]	990<br>[613, 1599]	<0.001
**MCP-1**	1553<br>[1169, 2063]	2954<br>[2164, 4033]	1808<br>[1195, 2734]	6083<br>[4197, 8816]	4641<br>[3328, 6473]	<0.001
**MIP-1a**	65<br>[49, 87]	44<br>[33, 59]	34<br>[25, 46]	89<br>[62, 127]	183<br>[130, 256]	<0.001
**MIP-1b**	271<br>[206, 355]	351<br>[268, 458]	278<br>[211, 365]	763<br>[544, 1070]	1223<br>[888, 1685]	<0.001
**RANTES**	36<br>[29, 46]	26<br>[20, 32]	19<br>[15, 24]	26<br>[19, 36]	31<br>[23, 41]	<0.001
**SDF-1a**	1656<br>[1257, 2182]	1218<br>[929, 1596]	1184<br>[897, 1561]	2835<br>[1993, 4033]	3749<br>[2695, 5214]	<0.001
**Interferons**						
**IFNa**	0.13<br>[0.03, 0.23]	0.08<br>[0.01, 0.18]	0.02<br>[0.06, 0.12]	0.01<br>[0.10, 0.13]	0.20<br>[0.07, 0.33]	0.113
**IFNg**	31<br>[21, 46]	57<br>[39, 83]	53<br>[36, 78]	112<br>[73, 171]	114<br>[76, 173]	<0.001
**Tumor necrosis factors**						
**TNFa**	19<br>[14, 25]	27<br>[20, 35]	21<br>[16, 28]	42<br>[30, 59]	48<br>[34, 66]	<0.001
**TNFb**	0.26<br>[0.06, 0.50]	0.09<br>[0.08, 0.30]	0.12<br>[0.06, 0.33]	0.03<br>[0.16, 0.25]	0.07<br>[0.11, 0.30]	0.096
**Colony stimulating factors**						
**GM-CSF**	13<br>[7, 24]	15<br>[8, 27]	8<br>[4, 15]	24<br>[12, 48]	32<br>[16, 62]	<0.001

Data are shown as average values and 95% confidence intervals (in brackets), estimated by linear mixed effect model for repeated measures. The p-values refer to the change over time and were assessed using ANOVA.

Supplementary Table 5. Cytokine levels assessed in perfusate samples at various time points during NMP (n=26 donor livers subjected to NMP): differences between donation after brain death (DBD, n=18) and donation after circulatory death (DCD, n=8).

Cytokine	1 h (pg/ml) average value [95% CI]	4 h (pg/ml) average value [95% CI]	6 h (pg/ml) average value [95% CI]	12 h (pg/ml) average value [95% CI]	24 h (pg/ml) average value [95% CI]	p-value
**Interleukins**						
**IL-1a**						
DBD	2 [1, 5]	8 [4, 15]	5 [2, 10]	9 [4, 18]	18 [9, 34]	0.661
DCD	2 [0, 5]	4 [1, 12]	5 [1, 14]	13 [4, 34]	15 [5, 39]	
**IL-1b**						
DBD	8 [4, 13]	12 [7, 21]	11 [6, 19]	14 [7, 26]	14 [7, 26]	0.490
DCD	8 [3, 18]	15 [7, 34]	19 [7, 45]	19 [7, 50]	19 [7, 50]	
**IL-2**						
DBD	5 [2, 9]	8 [4, 14]	7 [4, 13]	11 [5, 23]	17 [8, 32]	0.549
DCD	3 [1, 9]	11 [4, 26]	12 [5, 32]	21 [7, 55]	28 [10, 74]	
**IL-4**						
DBD	12 [7, 19]	40 [25, 64]	27 [17, 43]	32 [18, 58]	57 [33, 97]	0.868
DCD	10 [5, 21]	35 [17, 69]	32 [14, 69]	60 [26, 138]	64 [28, 146]	
**IL-5**						
DBD	6 [2, 13]	3 [1, 8]	2 [0, 5]	1 [0, 4]	2 [0, 6]	0.182
DCD	7 [2, 23]	8 [2, 25]	5 [1, 19]	4 [0, 15]	7 [1, 27]	
**IL-6**						
DBD	659 [238, 1820]	765 [268, 2176]	289 [103, 805]	285 [72, 1123]	741 [212, 2577]	0.050
DCD	728 [149, 3528]	1087 [198, 5948]	1032 [161, 6592]	3915 [612, 25037]	8204 [1282, 52451]	
**IL-7**						
DBD	5 [4, 6]	8 [6, 10]	7 [6, 9]	18 [13, 24]	27 [21, 35]	0.103
DCD	4 [3, 6]	7 [5, 10]	9 [6, 13]	42 [28, 62]	99 [66, 147]	
**IL-9**						
DBD	12 [3, 19]	42 [35, 50]	31 [23, 38]	53 [43, 64]	64 [55, 74]	0.990
DCD	16	36	33	61	56	

	[4, 27]	[25, 48]	[20, 47]	[46, 75]	[42, 71]	
**IL-10**						
DBD	845 [424, 1683]	912 [457, 1817]	324 [160, 655]	131 [59, 287]	187 [88, 397]	0.687
DCD	646 [215, 1938]	646 [215, 1938]	498 [107, 2292]	1123 [352, 3576]	341 [107, 1089]	
**IL12p70**						
DBD	2.8 [2.2, 3.5]	3.9 [2.2, 3.5]	3.9 [3.2, 4.7]	2.2 [1.6, 2.9]	2.2 [1.6, 2.8]	0.677
DCD	2.3 [1.6, 3.2]	4.4 [3.2, 5.8]	3.9 [2.7, 5.3]	1.7 [1, 2.6]	2.4 [1.5, 3.6]	
**IL-13**						
DBD	8 [5, 10]	8 [6, 11]	7 [5, 9]	12 [9, 18]	15 [11, 21]	0.548
DCD	6 [4, 10]	6 [4, 10]	6 [3, 10]	15 [9, 24]	15 [9, 26]	
**IL-15**						
DBD	0.86 [0.38, 1.51]	0.52 [0.13, 1.03]	0.22 [0.08, 0.63]	0.11 [0.22, 0.59]	0.11 [0.21, 0.55]	0.820
DCD	0.82 [0.17, 1.82]	0.57 [0.01, 1.43]	0.36 [0.17, 1.21]	0.46 [0.13, 1.46]	0.02 [0.39, 0.72]	
**IL-17a**						
DBD	2.35 [1.17, 4.18]	2.85 [1.51, 4.90]	2.45 [1.26, 4.28]	5.27 [2.80, 9.35]	6.26 [3.51, 10.68]	0.657
DCD	1.56 [0.35, 3.86]	2.17 [0.67, 5.03]	1.71 [0.35, 4.42]	5.86 [2.30, 13.25]	7.07 [2.89, 15.75]	
**IL-18**						
DBD	401 [265, 607]	573 [379, 864]	490 [325, 739]	730 [467, 1141]	819 [531, 1265]	0.830
DCD	452 [243, 838]	503 [271, 933]	511 [268, 972]	567 [293, 1098]	568 [293, 1100]	
**IL-21**						
DBD	7 [2, 20]	3 [1, 10]	2 [0, 6]	3 [0, 10]	4 [1, 13]	0.802
DCD	7 [1, 34]	6 [1, 28]	1 [1, 8]	8 [1, 41]	5 [0, 29]	
**IL-22**						
DBD	16 [5, 45]	21 [7, 57]	17 [6, 46]	11 [3, 37]	24 [7, 72]	0.314
DCD	6 [1, 27]	16 [3, 73]	8 [1, 43]	3 [0, 20]	6 [0, 34]	
**IL-23**						
DBD	0.47 [0.01, 1.17]	0.91 [0.30, 1.81]	1.92 [1.00, 3.25]	0.14 [0.30, 0.87]	0.38 [0.12, 1.18]	0.484
DCD	0.52 [0.15, 1.70]	0.87 [0.05, 2.33]	0.73 [0.10, 2.32]	0.02 [0.52, 0.98]	0.02 [0.52, 0.98]	
**IL-27**						
DBD	5 [2, 10]	14 [7, 27]	15 [8, 27]	30 [14, 66]	35 [17, 72]	0.427
DCD	4	9	9	36	35	

	[0, 7]	[3, 23]	[3, 27]	[11, 109]	[11, 105]	
**IL-31**						
DBD	0.54 [0.20, 0.98]	0.13 [0.12, 0.44]	0.04 [0.18, 0.33]	0.01 [0.28, 0.37]	0.06 [0.21, 0.43]	0.942
DCD	0.57 [0.09, 1.26]	0.10 [0.24, 0.58]	0.02 [0.36, 0.48]	0.02 [0.38, 0.54]	0.12 [0.29, 0.77]	
**Chemokines**						
**Eotaxin**						
DBD	39 [24, 63]	34 [21, 55]	27 [17, 43]	71 [39, 129]	100 [57, 173]	0.481
DCD	38 [9, 38]	17 [8, 35]	17 [7, 39]	111 [47, 261]	353 [150, 828]	
**GROa**						
DBD	17 [9, 32]	185 [101, 336]	76 [41, 140]	154 [73, 320]	474 [239, 936]	0.390
DCD	19 [7, 47]	152 [63, 366]	181 [63, 518]	606 [211, 1737]	651 [227, 1863]	
**IL-8**						
DBD	243 [143, 411]	2478 [1391, 4415]	1424 [660, 3070]	3945 [1844, 8441]	5271 [2751, 10099]	0.753
DCD	288 [133, 619]	1241 [472, 3260]	1077 [126, 9124]	5347 [2035, 14044]	4297 [1635, 11286]	
**IP-10**						
DBD	132 [79, 218]	239 [146, 391]	147 [89, 241]	591 [318, 1099]	900 [506, 1602]	0.724
DCD	109 [52, 229]	202 [96, 422]	294 [128, 673]	738 [302, 1799]	1280 [525, 3120]	
**MCP-1**						
DBD	1585 [1128, 2227]	3656 [2556, 5231]	1890 [1189, 3003]	6191 [3893, 9845]	5071 [3426, 7504]	0.219
DCD	1481 [899, 2438]	1567 [853, 2875]	1614 [659, 3950]	5505 [2994, 10121]	3672 [1997, 6751]	
**MIP-1a**						
DBD	69 [49, 99]	51 [36, 72]	39 [27, 55]	89 [58, 137]	150 [101, 225]	0.483
DCD	57 [34, 97]	33 [19, 56]	24 [13, 43]	88 [47, 163]	289 [155, 535]	
**MIP-1b**						
DBD	294 [210, 411]	382 [275, 529]	272 [197, 375]	675 [445, 1024]	1128 [767, 1658]	0.901
DCD	227 [139, 371]	290 [178, 473]	308 [177, 536]	979 [539, 1777]	1498 [825, 2719]	
**RANTES**						
DBD	36 [27, 47]	27 [20, 35]	18 [13, 23]	23 [16, 33]	26 [19, 36]	0.287
DCD	38 [25, 58]	23 [15, 36]	25 [15, 40]	36 [21, 60]	48 [29, 81]	
**SDF-1a**						
DBD	1632	1151	1178	2548	3439	0.410

	[1150, 2314]	[820, 1615]	[847, 1639]	[1626, 3992]	[2289, 5165]	
DCD	1708 [1058, 2757]	1364 [845, 2202]	1201 [691, 2085]	3402 [1860, 6220]	4458 [2438, 8149]	
**Interferons**						
**IFNa**						
DBD	0.07 [0.04, 0.19]	0.08 [0.03, 0.20]	0.02 [0.08, 0.13]	0.00 [0.13, 0.15]	0.27 [0.11, 0.44]	0.704
DCD	0.26 [0.08, 0.47]	0.09 [0.07, 0.27]	0.04 [0.13, 0.25]	0.02 [0.17, 0.24]	0.04 [0.14, 0.27]	
**IFNg**						
DBD	32 [20, 51]	66 [42, 103]	57 [36, 89]	131 [78, 220]	132 [80, 217]	0.357
DCD	29 [14, 57]	41 [21, 81]	46 [22, 95]	80 [37, 172]	82 [38, 176]	
**Tumor necrosis factors**						
**TNFa**						
DBD	18 [13, 26]	26 [19, 37]	20 [14, 28]	39 [25, 60]	45 [30, 67]	0.525
DCD	20 [12, 34]	28 [17, 46]	24 [13, 42]	49 [26, 90]	56 [30, 104]	
**TNFb**						
DBD	0.29 [0.04, 0.60]	0.13 [0.08, 0.40]	0.17 [0.05, 0.45]	0.06 [0.17, 0.35]	0.12 [0.11, 0.41]	0.476
DCD	0.20 [0.12, 0.65]	0.00 [0.27, 0.37]	0.02 [0.31, 0.37]	0.04 [0.33, 0.37]	0.04 [0.33, 0.37]	
**Colony stimulating factors**						
**GM-CSF**						
DBD	14 [6, 30]	16 [7, 33]	8 [4, 17]	19 [8, 46]	30 [13, 68]	0.949
DCD	10 [3, 32]	12 [4, 37]	8 [2, 29]	36 [10, 124]	36 [10, 123]	

Data are shown as average values and 95% confidence intervals (in brackets), estimated by linear mixed effect model for repeated measures. The p-values refer to the difference between DBD and DCD and were assessed using ANOVA.

Supplementary Table 6. Cytokine levels assessed in perfusate samples at various time points during NMP (n=26 donor livers subjected to NMP): differences between transplanted (n=18) and discarded (n=8) livers.

Cytokine	1 h (pg/ml) average value [95% CI]	4 h (pg/ml) average value [95% CI]	6 h (pg/ml) average value [95% CI]	12 h (pg/ml) average value [95% CI]	24 h (pg/ml) average value [95% CI]	p-value
**Interleukins**						
**IL-1a**						
Transplanted	2 [1, 5]	7 [4, 14]	5 [2, 9]	8 [4, 16]	16 [8, 32]	0.903
Discarded	1 [0, 4]	5 [2, 14]	6 [2, 15]	14 [5, 35]	17 [6, 42]	
**IL-1b**						
Transplanted	6 [3, 11]	12 [7, 20]	11 [6, 19]	10 [5, 20]	10 [5, 20]	0.092
Discarded	12 [5, 26]	16 [7, 35]	18 [7, 40]	29 [12, 68]	27 [12, 62]	
**IL-2**						
Transplanted	4 [2, 8]	7 [4, 12]	7 [4, 12]	10 [5, 21]	13 [6, 26]	0.114
Discarded	5 [2, 11]	15 [6, 34]	14 [5, 33]	24 [9, 59]	36 [15, 83]	
**IL-4**						
Transplanted	10 [6, 17]	36 [23, 57]	25 [16, 40]	30 [16, 54]	48 [27, 86]	0.213
Discarded	14 [7, 29]	44 [22, 86]	35 [17, 72]	64 [30, 137]	82 [40, 166]	
**IL-5**						
Transplanted	4 [2, 10]	3 [1, 7]	2 [0, 5]	1 [0, 5]	2 [0, 7]	0.123
Discarded	13 [4, 39]	9 [2, 28]	5 [1, 18]	2 [0, 10]	4 [1, 15]	
**IL-6**						
Transplanted	651 [241, 1761]	823 [312, 2170]	327 [120, 892]	249 [65, 948]	392 [103, 1488]	0.032
Discarded	755 [160, 3536]	770 [103, 5735]	725 [118, 4449]	4478 [730, 27437]	9816 [2086, 46166]	
**IL-7**						
Transplanted	4 [3, 6]	8 [6, 10]	8 [6, 11]	22 [15, 31]	31 [22, 43]	0.447
Discarded	5 [3, 8]	8 [5, 13]	7 [4, 11]	26 [16, 40]	57 [38, 86]	
**IL-9**						
Transplanted	10 [2, 18]	39 [31, 46]	29 [21, 37]	54 [43, 65]	62 [52, 73]	0.178
Discarded	18 [7, 30]	45 [33, 57]	36 [24, 49]	58 [45, 72]	62 [49, 75]	

**IL-10**						
Transplanted	762 [379, 1531]	808 [401, 1628]	302 [145, 627]	137 [59, 317]	141 [62, 317]	0.363
Discarded	699 [248, 1964]	824 [270, 2510]	437 [111, 1720]	696 [228, 2124]	424 [145, 1236]	
**IL12p70**						
Transplanted	2.78 [2.22, 3.44]	2.93 [2.37, 3.60]	3.89 [3.18, 4.72]	1.98 [1.39, 2.71]	1.88 [1.34, 2.54]	0.192
Discarded	2.45 [1.73, 3.36]	4.26 [3.17, 5.65]	3.93 [2.84, 5.32]	2.06 [1.34, 3.01]	2.87 [2.02, 3.97]	
**IL-13**						
Transplanted	7 [5, 10]	8 [6, 11]	7 [5, 9]	12 [9, 18]	12 [8, 17]	0.807
Discarded	7 [4, 11]	7 [4, 11]	6 [4, 10]	14 [9, 22]	21 [13, 33]	
**IL-15**						
Transplanted	0.61 [0.20, 1.16]	0.52 [0.13, 1.03]	0.20 [0.11, 0.60]	0.20 [0.17, 0.74]	0.06 [0.26, 0.51]	0.396
Discarded	1.49 [0.61, 2.84]	0.58 [0.02, 1.43]	0.40 [0.11, 1.21]	0.29 [0.20, 1.08]	0.16 [0.26, 0.83]	
**IL-17a**						
Transplanted	2.15 [1.06, 3.83]	2.44 [1.26, 4.25]	2.02 [0.98, 3.62]	4.06 [2.05, 7.42]	4.64 [2.44, 8.23]	0.412
Discarded	1.97 [0.58, 4.59]	3.08 [1.17, 6.67]	2.73 [0.94, 6.18]	8.56 [3.85, 17.84]	10.46 [4.96, 21.04]	
**IL-18**						
Transplanted	343 [232, 507]	464 [315, 684]	395 [268, 584]	571 [369, 884]	666 [434, 1021]	0.068
Discarded	649 [363, 1162]	807 [451,1444]	838 [463, 1517]	996 [543, 1827]	994 [549, 1800]	
**IL-21**						
Transplanted	6 [2, 18]	3 [1, 10]	2 [0, 7]	3 [0, 11]	3 [1, 12]	0.736
Discarded	9 [1, 42]	5 [0, 27]	1 [1, 8]	6 [1, 32]	7 [1, 33]	
**IL-22**						
Transplanted	18 [6, 50]	22 [7, 60]	16 [5, 46]	5 [1, 20]	14 4, 45]	0.613
Discarded	5 [0, 24]	15 [3, 69]	9 [1, 45]	11 [1, 57]	17 [3, 81]	
**IL-23**						
Transplanted	0.19 [0.19, 0.75]	0.82 [0.25, 1.65]	1.78 [0.90, 3.05]	0 [0.40, 0.65]	0.53 [0.05, 1.48]	0.679
Discarded	1.38 [0.35, 3.17]	1.09 [0.19, 2.66]	0.29 [0.32, 1.42]	0.29 [0.32, 1.42]	0.04 [0.47, 0.74]	
**IL-27**						
Transplanted	4 [2, 8]	12 [6, 22]	13 [6, 24]	29 [12, 66]	28 [13, 61]	0.707
Discarded	4 [1, 10]	14 [5, 36]	14 [5, 37]	38 [13, 108]	48 [18, 128]	

**IL-31**						
Transplanted	0.29 [0.02, 0.65]	0.09 [0.14, 0.38]	0.04 [0.18, 0.32]	0.02 [0.27, 0.41]	0.02 [0.25, 0.39]	0.214
Discarded	1.29 [0.61, 2.26]	0.19 [0.16, 0.69]	0.02 [0.33, 0.43]	0.04 [0.36, 0.43]	0.19 [0.18, 0.74]	
**Chemokines**						
**Eotaxin**						
Transplanted	37 [23, 59]	27 [17, 43]	22 [13, 34]	47 [26, 87]	68 [38, 121]	0.161
Discarded	21 [10, 41]	28 [14, 55]	31 [15, 63]	184 [86, 392]	416 [204, 847]	
**GROa**						
Transplanted	16 [8, 29]	158 [88, 282]	75 [41, 137]	129 [60, 277]	400 [192, 829]	0.084
Discarded	24 [10, 59]	209 [83, 519]	163 [57, 462]	648 [246, 1704]	813 [327, 2023]	
**IL-8**						
Transplanted	222 [131, 375]	1746 [983, 3100]	1376 [641, 2953]	3772 [1770, 8036]	5783 [2832, 11807]	0.332
Discarded	347 [162, 743]	3298 [1264, 8604]	1189 [142, 9934]	6029 [2311, 15723]	4194 [1860, 9453]	
**IP-10**						
Transplanted	124 [76, 204]	193 [119, 312]	135 [83, 220]	554 [292, 1053]	823 [445, 1519]	0.148
Discarded	124 [60, 256]	327 [158, 673]	348 [154, 782]	820 [364, 1844]	1411 [658, 3024]	
**MCP-1**						
Transplanted	1418 [1000, 2009]	2988 [2070, 4312]	1510 [918, 2482]	6203 [3869, 9947]	5352 [3477, 8237]	0.751
Discarded	1877 [1125, 3131]	2853 [1542, 5279]	2776 [1305, 5906]	6234 [3369, 11532]	3922 [2284, 6733]	
**MIP-1a**						
Transplanted	64 [45, 92]	49 [34, 69]	35 [2, 50]	81 [51, 129]	144 [93, 223]	0.879
Discarded	67 [40, 114]	36 [21, 61]	32 [18, 56]	99 [55, 178]	252 [145, 436]	
**MIP-1b**						
Transplanted	312 [225, 435]	383 [277, 529]	256 [185, 355]	650 [423, 999]	1084 [719, 1634]	0.932
Discarded	200 [123, 325]	288 [177, 467]	348 [209, 580]	947 [550, 1629]	1419 [851, 2363]	
**RANTES**						
Transplanted	34 [25, 45]	25 [19, 34]	18 [14, 24]	24 [16, 35]	25 [17, 36]	0.215
Discarded	43 [28, 66]	26 [17, 40]	21 [13, 34]	31 [19, 51]	44 [28, 68]	
**SDF-1a**						
Transplanted	1806 [1274, 2562]	1160 [827, 1627]	1180 [840, 1658]	2735 [1700, 4401]	3457 [2207, 5416]	0.905

Discarded	1411 [874, 2 277]	1343 [832, 2 167]	1200 [719, 2 001]	2932 [1690, 5 088]	4104 [2462, 68 42]	
**Interferons**						
**IFNa**						
Transplanted	0.04 [0.07, 0.15]	0.35 [0.16, 0.5]	0.03 [0.07, 0.14]	0.00 [0.13, 0.16]	0.18 [0.03, 0.35]	0.086
Discarded	0.06 [0.04, 0.18]	0.12 [0.04, 0.31]	0.01 [0.14, 0.19]	0.01 [0.16, 0.21]	0.22 [0.03, 0.44]	
**IFNg**						
Transplanted	29 [18, 45]	49 [32, 77]	43 [27, 67]	85 [50, 145]	100 [60, 167]	0.140
Discarded	38 [19, 73]	78 [40, 151]	90 [45, 178]	187 [92, 378]	155 [78, 306]	
**Tumor necrosis factors**						
**TNFa**						
Transplanted	17 [12, 24]	24 [17, 33]	18 [13, 25]	35 [22, 54]	39 [26, 59]	0.012
Discarded	24 [15, 39]	35 [22, 57]	31 [19, 52]	60 [35, 103]	69 [41, 114]	
**TNFb**						
Transplanted	0.16 [0.07, 0.43]	0.13 [0.09, 0.39]	0.17 [0.05, 0.45]	0.08 [0.15, 0.38]	0.09 [0.15, 0.38]	0.910
Discarded	0.52 [0.10, 1.08]	0.01 [0.26, 0.39]	0.03 [0.30, 0.35]	0.05 [0.32, 0.33]	0.05 [0.24, 0.46]	
**Colony stimulating factors**						
**GM-CSF**						
Transplanted	11 [5, 24]	13 [6, 26]	7 [3, 14]	16 [6, 40]	17 [7, 41]	0.180
Discarded	17 [5, 48]	20 [6, 57]	12 [4, 37]	45 [14, 141]	81 [27, 237]	

Data are shown as average values and 95% confidence intervals (in brackets), estimated by linear mixed effect model for repeated measures. The p-values refer to the difference between transplanted and discarded livers and were assessed using ANOVA.

Supplementary Table 7. Overview of all antibodies - clones, providers, dilutions, antigen retrieval (AR) and Opal fluorophore pairing used for multispectral imaging

Antibody	Clone	Provider	Dilution	pH (AR)	Opal Pairing
**Panel 1. Multiplex imaging – phenotyping of immune cells in liver tissue**					
CD15	MMA	Abcam	1:100	6	520
CD20	L26	Dako	1:200	6	540
CD8	C8\144B	Dako	1:200	9	570
CD3	polyclonal	Dako	1:250	6	620
CD68	PG-M1	Dako	1:200	9	650
Cytokeratin	AE1/AE3<br>C-11	Dako<br>Abcam	1:500<br>1:1000	9	690
DAPI	-	Akoya Biosciences	1:15	7.4	450
**Panel 2. CD15 CXCR2 double staining**					
CXCR2	EPR22301-103	Abcam	1:1000	9	520
CD15	MMA	Abcam	1:100	6	570
DAPI	-	Akoya Biosciences	1:15	7.4	450

Supplementary Table 8. Antibodies used for flow cytometry in liver tissue

Antibody	Clone	Conjugate	Dilution	Company
CD34	HPCA-2	FITC	1:20	BDBiosciences
7AAD	-	BB700	1:14	BDBiosciences
CD90	5E10	APC	1:100	Biolegend
CD19	HI98	APC-R700	1:400	BDBiosciences
CD16	eBioCB16	APC-eF780	1:25	Thermo
CD56	NCAM16.2	BUV395	1:100	BDBiosciences
CD3	UCHT	BUV496	1:400	BDBiosciences
CD8	RPA-T8	BUV563	1:200	BDBiosciences
CD28	CD28.2	BUV615P	1:500	BDBiosciences
CD4	SK3	BUV737	1:200	BDBiosciences
CD45	HI30	BUV805	1:100	BDBiosciences
CD38	HIT2	BV421	1:200	BDBiosciences
HLA-DR	G46-6	BV480	1:100	BDBiosciences
CD31	WM59	BV605	1:100	Biolegend
CD123	7G3	BV650	1:200	BDBiosciences
CD14	MφP9	BV711	1:200	BDBiosciences
CD15	HI98	BV786	1:100	BDBiosciences
CD161	DX12	PE	1:40	BDBiosciences
CD193	5E8	PE-CF594	1:167	BDBiosciences
CD326	9C4	PE-Cy7	1:2000	Biolegend

Supplementary Table 9. Antibodies used for flow cytometry in perfusate

Antibody	Clone	Conjugate	Dilution	Company
CD1c	F10/21A3	BV421	1:20	BDBiosciences
CD3	HIT3a	BV510<br>FITC	1:40<br>1:20	BDBiosciences
CD4	RPA-T4, SK3	PE-CF594<br>BV786	1:100<br>1:100	BDBiosciences
CD8	Hit8a, RPA-T8	FITC<br>PE-CF594<br>AF700	1:200<br>1:250<br>1:50	BDBiosciences
CD11b	WM59	PE-Cy7	1:250	BDBiosciences
CD11c	B-ly6	BV605	1:50	BDBiosciences
CD14	MΨP9, M5E2	BV711<br>FITC<br>BV785	1:100<br>1:20<br>1:20	BDBiosciences
CD15	HI98	PE-CF594	1:400	BDBiosciences
CD16	3G8	PE<br>BV711	1:200<br>1:100	BDBiosciences
CD19	Hib19, SJ25C1	PE-Cy7<br>BV605<br>FITC	1:100<br>1:100<br>1:20	ThermoFisher, BD
CD20	2H7	FITC	1:20	BDBiosciences
CD21	B-ly4	PE	1:20	BDBiosciences
CD24	ML5	PE-Cy7	1:100	BDBiosciences
CD25	BC96	BV605	1:33	BioLegend
CD27	M-T271	PE<br>APC	1:66<br>1:20	BDBiosciences
CD28	CD28.2	APC	1:200	BDBiosciences
CD31	WM59	BV650	1:2000	BDBiosciences
CD38	HIT2	BV786	1:100	BDBiosciences
CD45	2D1	APC-H7	1:100	BDBiosciences
CD45RA	HI100	BB515	1:100	BDBiosciences
CD45RO	UCHL1	BV650<br>APC	1:500<br>1:40	BDBiosciences
CD56	NCAM162, B159	APC<br>FITC	1:50<br>1:100	BDBiosciences
CD57	NK-1	PE-CF594	1:100	BDBiosciences
CD64	10.1	BV421<br>BV605	1:100<br>1:100	ThermoFisher
CD66b	G10F5	FITC	1:20	BDBiosciences
CD69	FN50	BV786	1:100	BDBiosciences
CD86	2331	PE-Cy7	1:100	BDBiosciences
CD103	Ber-Act8	PerCP-ef710<br>BV605	1:100<br>1:33	ThermoFisher, BD
CD127	HIL-7R-M21	PE	1:50	BDBiosciences
CD123	9F5	BV510<br>PE	1:100<br>1:100	BDBiosciences
CD141	AD5-14H12	PE	1:20	Miltenyi
CD161	DX12	APC	1:10	BDBioscience
CD163	GHI/61	BV421	1:50	BDBioscience

CD303	AC144	APC	1:50	Miltenyi
CD314	BAT221	PE	1:20	Miltenyi
CD335	9E2	BV421	1:100	BDBioscience
CCR7	150503	BV421	1:33	BDBiosciences
CXCR6	13B1ES	BV650	1:50	BDBiosciences
FoxP3	PCH101	APC	1:20	ThermoFisher
HLA-DR	LN3, G46-6	AF700<br>BV510<br>BV786<br>FITC	1:33<br>1:250<br>1:100<br>1:50	BDBiosciences
IgD	IA6-2	BB515	1:100	BDBiosciences
IgM	G20-127	BV421	1:100	BDBiosciences
Siglec8	7C9	APC	1:50	BioLegend
TCRVα7.2	3C10	PE	1:50	BioLegend
αβ TCR	T10B9.1.A-31	FITC	1:20	BDBioscience
γδ TCR	11F2	BV650	1:50	BDBioscience
