# Supplementary material for: Immune cell dynamics deconvoluted by single-cell RNA sequencing in normothermic machine perfusion of the liver
Source: Nat Commun. 2023 Apr 21;14:2285. doi: 10.1038/s41467-023-37674-8 (PMC10121614; doi:10.1038/s41467-023-37674-8)
Supplement: Supplementary file 3 — Reporting Summary [file 41467_2023_37674_MOESM3_ESM.pdf]

nature portfolio

Corresponding author(s): Stefan Schneeberger, Dominik Wolf

Last updated by author(s): Feb 27, 2023

Reporting Summary

Nature Portfolio wishes to improve the reproducibility of the work that we publish. This form provides structure for consistency and transparency in reporting. For further information on Nature Portfolio policies, see our Editorial Policies and the Editorial Policy Checklist.

Statistics

For all statistical analyses, confirm that the following items are present in the figure legend, table legend, main text, or Methods section.

n/a	Confirmed	
☐	☒	The exact sample size (*n*) for each experimental group/condition, given as a discrete number and unit of measurement
☐	☒	A statement on whether measurements were taken from distinct samples or whether the same sample was measured repeatedly
☐	☒	The statistical test(s) used AND whether they are one- or two-sided<br>*Only common tests should be described solely by name; describe more complex techniques in the Methods section.*
☐	☒	A description of all covariates tested
☐	☒	A description of any assumptions or corrections, such as tests of normality and adjustment for multiple comparisons
☐	☒	A full description of the statistical parameters including central tendency (e.g. means) or other basic estimates (e.g. regression coefficient) AND variation (e.g. standard deviation) or associated estimates of uncertainty (e.g. confidence intervals)
☐	☒	For null hypothesis testing, the test statistic (e.g. *F*, *t*, *r*) with confidence intervals, effect sizes, degrees of freedom and *P* value noted<br>*Give P values as exact values whenever suitable.*
☒	☐	For Bayesian analysis, information on the choice of priors and Markov chain Monte Carlo settings
☒	☐	For hierarchical and complex designs, identification of the appropriate level for tests and full reporting of outcomes
☒	☐	Estimates of effect sizes (e.g. Cohen's *d*, Pearson's *r*), indicating how they were calculated

Our web collection on statistics for biologists contains articles on many of the points above.

Software and code

Policy information about availability of computer code

Data collection	Of 34 donor livers subjected to NMP, scRNASeq analysis was performed in eight randomly selected study livers. Therefore, wedge liver biopsy samples were collected at three individual time-points, i.e. pre NMP (T0), at end of NMP (T1), and after reperfusion (T2) if transplanted. Serial perfusate samples of another 26 study livers were collected at 1h, 4h, 6h, 12h and at end of NMP for cell quantification/phenotyping and cytokine assessment by flow cytometry and Luminex analysis.<br>Cell viability was measured with the BD Rhapsody scRNASeq platform (BD Biosciences).<br>Sequencing was performed on the NovaSeq 6000 System platform (Illumina).<br>The immunohistochemical staining was performed using an automated system (BOND-RX; Leica Biosystems).<br>Cells isolated from liver biopsies were measured on a FACSymphony A5 flow cytometer for FACS analysis.<br>Cytokine/chemokine protein levels were measured using the Cytokine&Chemokine 34-Plex Human ProcartaPlex Panel 1A (EPX340-12167-901, Thermo Fisher Scientific) in a Luminex MAGPIX instrument (Luminex Corporation)<br>Multiplex immunofluorescence staining slides were scanned using Mantra 2 Quantitative Pathology Workstation (Akoya Biosciences) and the Mantra Snap software v1.0.4. S
Data analysis	Bioinformatic pre-processing of the obtained FastQ sequencing files was performed via the cloud-based Seven Bridges Platform environment (Seven Bridges Genomics) using the BD Rhapsody WTA Analysis Pipeline app. Data was loaded into AnnData for further processing with scverse tools. Quality control was performed using scanpy.<br>Cell transcriptomes were embedded into a batch-corrected low-dimensional latent space using scVI, treating each sample as a separate batch.

Doublets were identified and removed using SOLO.
We performed transcription factor analysis with DoROthEA.
We used the CellChat database as obtained from omnipathdb to investigate differences in cell-to-cell communication between timepoints.
Spectral unmixing, multispectral image analysis and cell phenotyping was carried out using the inForm Tissue Analysis Software v2.4.10 (Akoya Biosciences).
Flow cytometry data were analyzed using FlowJo v10.7 software.
Cytokine/chemokine protein levels were analysed by xPonent 4.2 Rev.2 software (Luminex Corporation).
Analyses were performed using R statistical software (v4.0.3, Team RC, R Foundation for Statistical Computing) 98. Graphs were completed using GraphPad Prism 9.1.0 (216).

For manuscripts utilizing custom algorithms or software that are central to the research but not yet described in published literature, software must be made available to editors and reviewers. We strongly encourage code deposition in a community repository (e.g. GitHub). See the Nature Portfolio guidelines for submitting code & software for further information.

Data

Policy information about availability of data

All manuscripts must include a data availability statement. This statement should provide the following information, where applicable:

- Accession codes, unique identifiers, or web links for publicly available datasets
- A description of any restrictions on data availability
- For clinical datasets or third party data, please ensure that the statement adheres to our policy

Sequence data that support the findings of this study (all Figures) is available through the NCBI GEO accession GSE216584. Source data are provided with this paper. The source code to reproduce the data analysis is available from https://github.com/icbi-lab/nmp-liver. Processed input data and containerized software dependencies required to execute the code are available from zenodo: https://doi.org/10.5281/zenodo.7249006.

Human research participants

Policy information about studies involving human research participants and Sex and Gender in Research.

Reporting on sex and gender	An overview on the overall study population is presented in Table 1 (individual data are given in Supplementary Table 1). The findings were applied to both sex and/or gender. Sex and/or gender were determined on self-reporting.
Population characteristics	An overview on the overall study population is presented in Table 1 (individual data are given in Supplementary Table 1).
Recruitment	Between April 2019 and July 2021, a total of 34 donor livers subjected to normothermic machine perfusion were enrolled. All organs were accepted with the intent to transplant after NMP. There were no potential self-selelction bias.
Ethics oversight	The study was approved by the institutional review board of the Medical University of Innsbruck (protocol #1175/2018).

Note that full information on the approval of the study protocol must also be provided in the manuscript.

Field-specific reporting

Please select the one below that is the best fit for your research. If you are not sure, read the appropriate sections before making your selection.

[x] Life sciences [ ] Behavioural & social sciences [ ] Ecological, evolutionary & environmental sciences

For a reference copy of the document with all sections, see nature.com/documents/nr-reporting-summary-flat.pdf

Life sciences study design

All studies must disclose on these points even when the disclosure is negative.

Sample size	No sample size calculation was performed. Between April 2019 and July 2021 thirty-four donor livers were included. Liver biopsy samples were collected at three individual time-points, while serial perfusate samples were collected at five time points, generating a large data amounting, sufficient for the attempt to perform primarily a descriptive analysis (cell atlas).
Data exclusions	No data were excluded from the analysis.
Replication	All attempts at replication were successful (n=34).
Randomization	A randomization was not necessary in this study, because no groups were compared.
Blinding	Blinding was was not necessary in this study, because no groups were compared.

Reporting for specific materials, systems and methods

We require information from authors about some types of materials, experimental systems and methods used in many studies. Here, indicate whether each material, system or method listed is relevant to your study. If you are not sure if a list item applies to your research, read the appropriate section before selecting a response.

Materials & experimental systems

n/a	Involved in the study
☐	☒ Antibodies
☒	☐ Eukaryotic cell lines
☒	☐ Palaeontology and archaeology
☒	☐ Animals and other organisms
☒	☐ Clinical data
☒	☐ Dual use research of concern

Methods

n/a	Involved in the study
☒	☐ ChIP-seq
☐	☒ Flow cytometry
☒	☐ MRI-based neuroimaging

Antibodies

Antibodies used	An overview of all antibodies used in the study is given in the supplementary file.
Validation	An overview of all antibodies used in the study is given in the supplementary file.

Flow Cytometry

Plots

Confirm that:

☒ The axis labels state the marker and fluorochrome used (e.g. CD4-FITC).

☒ The axis scales are clearly visible. Include numbers along axes only for bottom left plot of group (a 'group' is an analysis of identical markers).

☒ All plots are contour plots with outliers or pseudocolor plots.

☒ A numerical value for number of cells or percentage (with statistics) is provided.

Methodology

Sample preparation

Serial perfusate samples of 26 livers subjected to NMP (5 ml) were collected in Cyto-Check BCT tubes (Streck) at 1h, 4h, 6h, 12h and at end of NMP. Immediate cell fixation in these tubes maintain cellular morphology and surface antigen expression and allows storage of samples at room temperature at least for five days. In our study fixation tubes were chosen to ease logistics. 500 µl of perfusate was used for every staining. Initially, red blood cells of 500 µl perfusate were lysed with the RBC Lysis Buffer (Thermo Scientific, eBioscience). After blocking with Fc Blocking Reagents (BD Bioscience) cells were incubated with the antibody master mixes in various combinations containing the antibodies. For the FoxP3 intracellular staining, cells were permeabilized using the Foxp3/Transcription Factor Staining Buffer Set (Thermo Scientific, ebioscience) and incubated with Normal Rat Serum (Thermo Scientific, ebioscience) prior to incubation with the FoxP3 antibody. F

Instrument

FACSymphony A5 flow cytometer.

Software

Data were analyzed using FlowJo v10.7 software.

Cell population abundance

For quantification of absolute cell numbers, BD Trucount Tubes (BD Bioscience) were used according to the manufacturer´s protocol and analysed using a LSRFortessa flow cytometer (Becton Dickinson and Company). Doublet exclusion was achieved by plotting forward and sideward scatter areas, heights, and widths (FSC- and SSC-A/-H/-W).

Gating strategy

Gating strategy for the general immune phenotype panel analysed in liver biopsies [n=3 donor livers at time points pre NMP (T0) and at the end of NMP (T1)]. In initial cleaning steps dead cells, debris and doublets were removed using 7-AAD staining and scatter characteristics. Leukocytes were then defined by CD45 staining end sequentially gated into granulocytes (upper left), macrophages/Kupffer cells (upper right), T cells (lower right) and B and NK cells (lower left).

Gating strategy for the general immune phenotype panel analysed in BD TruCount™ tubes (perfusate of n=26 donor livers subjected to NMP at various time points). Gate for beads were set on an ungated dot plot (FITC vs. PE). Duplets were removed using a FSC-A vs. FSC-H dot pot. Gates for lymphocytes and granulocates were set on a CD45 vs. SSC-A dot plot.

Gating and exclusion of all CD14+ monocytes were identified via CD14 versus CD64. The gated CD14+ monocytes were used to further discriminate different inflammatory/differentiation stages of monocytes (CD16 versus CD14) resulting in CD14+ +CD16- classical monocytes, CD14++CD16+ and CD14+ CD16++ monocytes, and CD16 vs CD64 to capture CD16+ CD64+ monocytes. Lymphocytes (CD14-CD64-) were gated on CD56+ NK cells and NKT cells (CD56+CD3+). NK cells were further subdivided into CD56 dim and CD56 high NK cells. Lymphocytes were devided into CD3+ T cells (CD56 vs. CD3) – gated T cells were used for identification of CD4+ T-cells and CD8+ T-cells (CD4 vs. CD8), and the gated lymphocytes were also used for

identification of the B cell population (CD19 vs CD3). The total numbers of events for each of these subsets and the number of beads in the TruCount™ tubes were used to calculate the concentration of the different subsets.

Gating strategy for the naive and memory subsets of T cells analysed in BD TruCount™ tubes (perfusate of n=26 donor livers subjected to NMP at various time points). Lymphocytes were gated in a FSC-a vs. SSC-A dot plot and duplets were removed using a FSC-A vs. FSC-H dot pot. CD45+ cells and CD3+ cells were gated versus SSC-A. CD3+ T cells were subdivides into CD4+ and CD8+ T-cells, double negative and double positive T cells. (CD4 vs. CD8). Naive T cells (CD45RA+, CCR7+), central memory T cells (CD45RA-, CCR7+) and effector memory T cells (CD45RA-, CCR7-) and TEMRA (CD45RA+, CCR7-) cells were determined.
Gating strategy for T cells subtypes analysed in BD TruCount™ tubes (perfusate of n=26 donor livers subjected to NMP at various time points). Lymphocytes were gated in a FSC-a vs. SSC-A dot plot and duplets were removed using a FSC-A vs. FSC-H dot pot. CD45+ cells were gated versus SSC-A. CD45+ cells were analysed for CD3 CD45RO expression and subdivided into CD4+, CD8+ double negative and double positive CD3+ T cells. Additionally, CD45+ cells were gated in CD3+ T cells vs. SSC-A. CD3+ T cells were subdivided into TCR αβ + and TCR γδ +T cells. These T cells subtypes were further divided into CD4+, CD8+, double negative and double positive T cells.

Gating strategy for mucosa associated invariant T cells (MAIT) and resident liver T cells analysed in BD TruCount™ tubes (perfusate of n=26 donor livers subjected to NMP at various time points). Lymphocytes were gated in a FSC-a vs. SSC-A dot plot and duplets were removed using a FSC-A vs. FSC-H dot pot. CD45+ cells were gated versus SSC-A. T cells were gated vs CD56. TCR Vα2.2+ CD161+ were defined as MAIT cells. CD3+ T cells were subdivided into CD4+ and CD8+ T cells. Resident CD8+ were defined as CD103+CD69+CXCR6+ cells. Gates of CXCR6 were set according to their FMOs. Resident CD4+ T cells were defined as HLA-DR+ cells.

Gating strategy for regulatory T cells analysed in BD TruCount™ tubes (perfusate of n=26 donor livers subjected to NMP at various time points). Lymphocytes were gated in a FSC-a vs. SSC-A dot plot and duplets were removed using a FSC-A vs. FSC-H dot pot. CD3+ cells were gated versus CD45+ cells. CD3+ T cells were subdivides into CD4+ cells (CD4+ vs. SSC-A). Regulatory T cells were determined as CD25+ and FoxP3+ CD127 dim cells.

Gating strategy for natural killer (NK) cells analysed in BD TruCount™ tubes (perfusate of n=26 donor livers subjected to NMP at various time points). Lymphocytes were gated in a FSC-a vs. SSC-A dot plot and duplets were removed using a FSC-A vs. FSC-H dot pot. CD45+ cells were gated versus SSC-A. CD45+ cells were further discriminated into CD56+ cells and linage - cells (CD3, CD14, CD19). CD56+ linage- cells were subdivided into CD56 bright and CD56 dim cells and in CD56+ CD16 -/dim and CD56+ CD16+ cells.

Gating strategy for granulocytes, hepatocytes and Kupffer cells analysed in BD TruCount™ tubes (perfusate of n=26 donor livers subjected to NMP at various time points). Granulocytes, hepatocytes and Kupffer cells were gated in a FSC-A vs. SSC-A dot plot and duplets were removed using a FSC-A vs. FSC-H dot pot. Hepatocytes were defined as big CD45- cells. Monocytes and macrophages were gated as CD14+CD64+ cells. CD163+CD64+ macrophages were subdivided from this population. Granulocytes were divided into HLA-DR low CD66b low and HLA-DR low and CD66b high cells. The latter were defined as eosinophils (Siglec-8+ CD16-). HLA-DR low CD66b low cells were divided into neutrophils (CD16+CD15+CD14-) and basophils (CD123+CD16+).

Gating strategy for dendritic cells (DCs) analysed in BD TruCount™ tubes (perfusate of n=26 donor livers subjected to NMP at various time points). Lymphocytes were gated in a FSC-a vs. SSC-A dot plot and duplets were removed using a FSC-A vs. FSC-H dot pot. CD45+ cells were gated versus SSC-A. CD45+ cells were further discriminated into HLA-DR+ cells and linage - cells (CD3, CD14, CD19, CD20). These cells were devided into HLA-DR+ CD11c- cells and HLA-DR+ CD11c+ cells. HLA-DR+ CD11c- cells were determined as plasmacytoide DCs (pDC) via CD303 and CD123. Activity of pDCs was analysed with CD86. HLA-DR+ CD11c+ DCs were subdivided into myleloid DC1 via CD141 and CD16 and myeloid DC2 via CD1c and CD141. Activity of subtypes of DCs was determined with CD86. Gates of CD86 were set according to their FMOs.

☒ Tick this box to confirm that a figure exemplifying the gating strategy is provided in the Supplementary Information.
